# A Preplanned Multi‐Stage Platform Trial for Discovering Multiple Superior Treatments With Control of FWER and Power

**DOI:** 10.1002/bimj.70025

**Published:** 2024-12-22

**Authors:** Peter Greenstreet, Thomas Jaki, Alun Bedding, Pavel Mozgunov

**Affiliations:** ^1^ Department of Mathematics and Statistics Lancaster University Lancaster UK; ^2^ Exeter Clinical Trials Unit University of Exeter Exeter UK; ^3^ MRC Biostatistics Unit University of Cambridge Cambridge UK; ^4^ Faculty for Informatics and Data Science University of Regensburg Regensburg Germany; ^5^ Data and Statistical Sciences Department Roche Products Ltd. Welwyn Garden City UK

**Keywords:** FWER, multi‐arm, multi‐stage, platform trials, preplanned

## Abstract

There is a growing interest in the implementation of platform trials, which provide the flexibility to incorporate new treatment arms during the trial and the ability to halt treatments early based on lack of benefit or observed superiority. In such trials, it can be important to ensure that error rates are controlled. This paper introduces a multi‐stage design that enables the addition of new treatment arms, at any point, in a preplanned manner within a platform trial, while still maintaining control over the family‐wise error rate. This paper focuses on finding the required sample size to achieve a desired level of statistical power when treatments are continued to be tested even after a superior treatment has already been found. This may be of interest if there are treatments from different sponsors which are also superior to the current control or multiple doses being tested. The calculations to determine the expected sample size is given. A motivating trial is presented in which the sample size of different configurations is studied. In addition, the approach is compared to running multiple separate trials and it is shown that in many scenarios if family‐wise error rate control is needed there may not be benefit in using a platform trial when comparing the sample size of the trial.

## 1 Introduction

Platform trials are a type of trial design which can aim to reduce the amount of time and cost of clinical trials, and in recent years, there has been an increase in the utilization of such trials, including during the COVID‐19 pandemic (Lee et al. 2021; Stallard et al. 2020). Clinical trials take many years to run and can cost billions of dollars (Mullard 2018). During this time, it is not uncommon for new promising treatments to emerge and become ready to join the current phase later (Choodari‐Oskooei et al. 2020). Therefore, it may be advantageous to include these treatments into an ongoing trial. This can have multiple potential benefits including: shared trial infrastructure; the possibility to use a shared control group; less administrative and logistical effort than setting up separate trials and enhance the recruitment (Burnett, König, and Jaki 2024; Meurer, Lewis, and Berry 2012). This results in useful therapies potentially being identified faster while reducing cost and time (Cohen et al. 2015).

There is an ongoing discussion about how to add new treatments to clinical trials (Cohen et al. 2015; Lee et al. 2021) in both a preplanned and in an unplanned manor (Burnett, König, and Jaki 2024; Greenstreet et al. 2024). In both Bennett and Mander (2020), Choodari‐Oskooei et al. (2020) approaches are proposed which extend the Dunnett test (Dunnett 1955) to allow for unplanned additional arms to be included in multi‐arm trials while still controlling the family‐wise error rate (FWER). This methodology does not incorporate the possibility of interim analyses.

FWER is often considered to be one of the strongest types of type I error control in a multi‐arm trial (Wason et al. 2016). There are other approaches one may wish to consider such as pairwise error rate (PWER) and the false discovery rate (FDR) (Bratton et al. 2016; Choodari‐Oskooei et al. 2020; Cui et al. 2023; Robertson et al. 2022). However, as discussed in Wason, Stecher, and Mander (2014), there are scenarios where FWER is seen as the recommended error control, and it can be a regulatory requirement.

One may wish to include interim analyses as they allow for ineffective treatments to be dropped for futility earlier and allow treatments to stop early if they are found superior to the control. Therefore, potentially improving the efficiency of the design of a clinical trial by decreasing the expected sample size and cost of a trial (Pocock 1977; Todd et al. 2001; Wason et al. 2016). Multi‐arm multi‐stage (MAMS) designs (Magirr, Jaki, and Whitehead 2012; Royston, Parmar, and Qian 2003) allow interim analyses while still allowing several treatments to be evaluated within one study, but do not allow for additional arms to be added throughout the trial. Burnett, König, and Jaki (2024) have developed an approach that builds on Hommel (2001) to incorporate unplanned additional treatment arms to be added to a trial already in progress using the conditional error principle (Proschan and Hunsberger 1995). This allows for modifications during the course of a trial. However, due to the unplanned nature of the adaptation, later treatments can be greatly underpowered compared to arms which begin the trial.

In a recent paper, Greenstreet et al. (2024) proposed a preplanned approach to adding additional arms in which interim analyses can be conducted and multiple arms can be evaluated with some arms being added at later time points. In this work, the trial was powered assuming that only one treatment may be taken forward. However, as discussed in the work by Urach and Posch (2016) and Serra, Mozgunov, and Jaki (2022), this may not always be the case. For example, one may be interested in: lower doses; multiple treatments from different sponsors; if another treatment has preferable secondary outcomes if it also meets the primary outcome. Furthermore, in Greenstreet et al. (2024), treatment arms can only be added when an interim analysis happens, this can greatly restrict when arms can join the trial. This results in potentially large time periods that a new treatment is available before able to join the trial, as it is waiting for an interim to be conducted.

In this work, we provide an analytical method for the adding of treatments at any point to a MAMS trial in a preplanned manner, while still controlling the statistical errors. The focus is on preplanned trials, so, at the design stage it is known how many treatments are likely to be added and at what point in the trial they are planned to be added. For example, this can happen in a pharmaceutical company when another treatment is looking promising but is in an earlier stage of development and is not yet ready to be evaluated in the planned trial but can be added later on. Due to the flexibility of the methodology, one can create multiple designs for different numbers of treatments and for each point the additional treatments may be added into the trial. As a result, one can present multiple options and then use the trial design which matches the reality of the trial.

The focus is on trials in which one is interested in continuing to investigate the other treatments even after a superior treatment has been found. In addition, multiple types of power will be considered, and will prove that the conjunctive power of the study is at its lowest for a given sample size when all the active treatments have a targeted clinically relevant effect, where the conjunctive power is the probability of finding all the active treatments with a clinically relevant effect.

This work will focus predominantly on the case where one has a fixed allocation ratio across all the treatments and the same number of interim analyses per treatment with the same boundary shape. The proposed methodology, however, is general, therefore can be implemented for when the allocation ratio between active treatments and control can be different between each stage and each arm. One needs to be cautious of the potential effects of time trends on the test statistics when changing allocation ratios between active treatments and the control treatment mid trial (Altman and Royston 1988; Getz and Campo 2017; Proschan and Evans 2020; Roig et al. 2023).

We begin by analytically calculating the FWER and power of the study and use these to calculate both the stopping boundaries and sample size. Then, in Section 2.4, the equations for sample size distribution and expected sample size are given. A trial example of FLAIR (Howard et al. 2021), in Section 3, is used to motivate a hypothetical trial of interest. The sample size and stopping boundaries are found for multiple types of power control and the effect of different treatment effects is studied. The trial designs are then compared to running multiple separate trials. Finally, in Section 4, there is a discussion of the paper.

## 2 Methodology

### 2.1 Setting

In the clinical trial design considered in this work, K experimental arms effectiveness is compared to a common control arm. The trial has $K^{★}$ treatments starting at the beginning of the trial, and the remaining $K-K^{★}$ treatments being added at later points into the platform. The primary outcome measured for each patient is assumed to be independent, continuous, and follows a normal distribution with a known variance ($\sigma^{2}$).

The points at which each active treatment arm is added are predetermined, but can be set to any point within the trial. Each of the K treatments is potentially tested at a series of analyses indexed by $j=1,\cdots ,J_{k}$, where $J_{k}$ is the maximum number of analyses for a given treatment k=1,⋯,K. Let n(k) denote the number of patients recruited to the control treatment before treatment k is added to the platform trial and define the vector of adding times by n(K)=(n(1),⋯,n(K)). Therefore, for treatments that start at the beginning of the trial, n(k)=0. We also denote $n_{k,j}$ as the number of patients recruited to treatment k by the end of its jth stage and define $n_{0,k,j}$ as the total number of patients recruited to the control at the end of treatment k’s jth stage. We define $n_{k}=n_{k,1}$ as the number recruited to the first stage of treatment k, k=1,⋯,K. The total sample size of a trial is denoted by N. The maximum total planned sample size is $\max\left(N\right)=\sum_{k=1}^{K}n_{k,J_{k}}+\max_{k\in 1,\cdots ,K}\left(n_{0,k,J_{k}}\right)$.

We define $r_{k,j}$ and $r_{0,k,j}$ as the ratio of the number of patients recruited to treatment k and to the control by treatment k’s jth stage, respectively, compared to the number of patients on treatment 1 at stage 1. Therefore, the ratio of the number of patients recruited on treatment 1 at stage 1 equals $r_{1,1}=1$. The relationship between $r_{k,j}$ and $n_{k,j}$ is $r_{k,j}=n_{k,j}/n_{1,1}$, the relationship between $r_{0,k,j}$ and $n_{0,k,j}$ is $r_{0,k,j}=n_{0,k,j}/n_{1,1}$. Also, r(k) denotes the ratio of the number of patients recruited to the control before treatment k is added to the trial compared to $r_{1,1}$. For example, if a trial was planned to have equal number of patients per arm per stage with active treatment 1 added at the beginning of the trial (r(1)=0) and a treatment, $k^{\prime }$, was added at the first interim then $r(k^{\prime })=1$ and at the first stage for $k^{\prime }$, $r_{0,k^{\prime },1}=2$. The ratio should be chosen to calculate the required boundaries to control the FWER as provided in Section 2.2.

Throughout the trial, the control arm is recruited and maintained for the entire duration. The comparisons between the control arm and the active treatment arms are based on concurrent controls, meaning that only participants recruited to the control arm at the same time as the corresponding active arm are used in the comparisons. Work on the use of nonconcurrent controls includes Lee and Wason (2020) and Marschner and Schou (2022).

The null hypotheses of interest are $H_{0k}:\mu_{k}\leq \mu_{0},k=1,\cdots ,K$, where $\mu_{1},\cdots ,\mu_{K}$ are the mean responses on the K experimental treatments and $\mu_{0}$ is the mean response of the control group. The global null hypothesis, $\mu_{0}=\mu_{1}=\mu_{2}=\cdots =\mu_{K}$ is denoted by $H_{G}$. At analysis j for treatment k, to test $H_{0k}$ it is assumed that responses, $X_{k,i}$, from patients $i=1,\cdots ,n_{k,j}$ are observed, as well as the responses $X_{0,i}$ from patients $i=n\left(k\right)+1,\cdots ,n_{0,k,j}$. These are the outcomes of the patients allocated to the control which have been recruited since treatment k has been added into the trial up to the jth analysis of treatment k. The null hypotheses are tested using the test statistics

$$Z_{k,j}=\frac{n_{k,j}^{-1}\sum_{i=1}^{n_{k,j}}X_{k,i}-{\left(n_{0,k,j}-n\left(k\right)\right)}^{-1}\sum_{i=n(k)+1}^{n_{0,k,j}}X_{0,i}}{\sigma \sqrt{{\left(n_{k,j}\right)}^{-1}+{\left(n_{0,k,j}-n\left(k\right)\right)}^{-1}}}.$$

The decision making for the trial is made by the upper and lower stopping boundaries, denoted as $U_{k}=\left(u_{k,1},\cdots ,u_{k,J_{k}}\right)$ and $L_{k}=\left(l_{k,1},\cdots ,l_{k,J_{k}}\right)$. These boundaries are utilized to determine whether to continue or halt a treatment arm or even the whole trial at various stages. The decision‐making process is as follows: if the test statistic for treatment k at stage j exceeds the upper boundary $u_{k,j}$, the null hypothesis $H_{0k}$ is rejected, and the treatment is stopped with the conclusion that it is superior to the control. Conversely, if $Z_{k,j}$ falls below the lower boundary $l_{k,j}$, treatment k is stopped for futility for all subsequent stages of the trial. If neither the superiority nor futility conditions are met, $l_{k,j}\leq Z_{k,j}\leq u_{k,j}$, treatment k proceeds to its next stage j+1. If all the active treatments are stopped, then the trial stops. The bounds are determined such that they control the FWER of the trial.

If one wants to change the allocation ratio between the control arm and active arms mid trial, then one should adjust the test statistics if there are any time trends present (Burnett, König, and Jaki 2024; Greenstreet et al. 2024; Lee and Wason 2020; Marschner and Schou 2022) otherwise the errors in the trial may be inflated (Proschan and Evans 2020; Roig et al. 2023). Changes to allocation ratios are out of the scope for this paper. Those adjustments would typically be adaptive in nature and may require adaptive design methodology for control of type I error. The notation defined throughout this section is provided in the Supporting Information (Section 1) in tables for reader's convenience.

### 2.2 FWER

The FWER in the strong sense is defined as

(1) $$\begin{array}{ccc} & & P(\text{reject at least one true}\;H_{0k}\;\text{under any null configuration},\\ & & \;k=1,\cdots ,K)\leq \alpha , \end{array}$$

where α is the desired level of control for the FWER. As proven in Greenstreet et al. (2024) which builds on Magirr, Jaki, and Whitehead (2012), if one can show for the given boundaries that the FWER is controlled at the desired level under the global null hypothesis then the FWER is controlled in the strong sense. The proof that this holds when continuing the trial even after finding a superior treatment, is provided in the Supporting Information (Section 2).

To calculate the FWER under the global null hypothesis, one needs to consider every possible outcome of the trial which results in no active treatments being declared superior to the control treatment. Each treatment can be stopped for futility at any of its stages ($1,\cdots ,J_{k}$); therefore, we define $j_{k}$ as the stage for treatment k where it stops. For treatment k to stop at stage $j_{k}$ for futility, the test statistics need to be within the following upper and lower boundaries $U_{k,j_{k}}\left(0\right)=\left(u_{k,1},\cdots ,l_{k,j_{k}}\right)$ and $L_{k,j_{k}}\left(0\right)=\left(l_{k,1},\cdots ,-\infty \right)$, respectively. To calculate the FWER, one needs to combine every possible combination of $j_{k}$ for all K treatments, so we define $j_{k}=\left(j_{1},\cdots ,j_{K}\right)$ as a list of the stages where each treatment stops. As each event that results in all the treatments being stopped for futility are disjoint, then the additivity probability theorem (Kolmogorov and Bharucha‐Reid 2018) can be used, which results in summing all the events. The FWER under the global null hypothesis then equals

(2) $$1-\sum_{\begin{array}{c} j_{k}=1\\ k=1,2,\text{…},K \end{array}}^{J_{k}}\Phi \left(L_{j_{k}}\left(0\right),U_{j_{k}}\left(0\right),\Sigma_{j_{k}}\right).$$

Here, Φ(L,U,Σ) denotes the multivariate normal distribution function with mean zero and covariance matrix Σ between the lower boundaries L and upper boundaries U. With $j_{k}$, one can define the vector of upper and lower limits for the multivariate standard normal distribution function as $U_{j_{k}}\left(0\right)=\left(U_{1,j_{1}}\left(0\right),\cdots ,U_{K,j_{K}}\left(0\right)\right)$ and $L_{j_{k}}\left(0\right)=\left(L_{1,j_{1}}\left(0\right),\cdots ,L_{K,j_{K}}\left(0\right)\right)$, which is the combination of required boundaries for each active treatment k given $j_{k}$.

The correlation matrix, $\Sigma_{j_{k}}$, complete correlation structure is

(3) $$\Sigma_{j_{k}}=\left(\begin{array}{ccccccc} \rho_{\left(1,1),(1,1\right)} & \rho_{\left(1,1),(1,2\right)} & \cdots & \rho_{\left(1,1\right),\left(1,j_{1}\right)} & \rho_{\left(1,1),(2,1\right)} & \cdots & \rho_{\left(1,1\right),\left(K,j_{k}\right)}\\ \rho_{\left(1,2),(1,1\right)} & \rho_{\left(1,2),(1,2\right)} & \cdots & \rho_{\left(1,2\right),\left(1,j_{1}\right)} & \rho_{\left(1,2),(2,1\right)} & \cdots & \rho_{\left(1,2\right),\left(K,j_{k}\right)}\\ \vdots & \vdots & \ddots & \vdots & \vdots & \ddots & \vdots \\ \rho_{\left(1,j_{1}\right),\left(1,1\right)} & \rho_{\left(1,j_{1}\right),\left(1,2\right)} & \cdots & \rho_{\left(1,j_{1}\right),\left(1,j_{1}\right)} & \rho_{\left(1,j_{1}\right),\left(2,1\right)} & \cdots & \rho_{\left(1,j_{1}\right),\left(K,j_{k}\right)}\\ \rho_{\left(2,1),(1,1\right)} & \rho_{\left(2,1),(1,2\right)} & \cdots & \rho_{\left(2,1\right),\left(1,j_{1}\right)} & \rho_{\left(2,1),(2,1\right)} & \cdots & \rho_{\left(2,1\right),\left(K,j_{k}\right)}\\ \vdots & \vdots & \ddots & \vdots & \vdots & \ddots & \vdots \\ \rho_{\left(K,j_{k}\right),\left(1,1\right)} & \rho_{\left(K,j_{k}\right),\left(1,2\right)} & \cdots & \rho_{\left(K,j_{k}\right),\left(1,j_{1}\right)} & \rho_{\left(K,j_{k}\right),\left(2,1\right)} & \cdots & \rho_{\left(K,j_{k}\right),\left(K,j_{k}\right)} \end{array}\right),$$

where $\rho_{\left(k,j\right),\left(k^{★},j^{★}\right)}$ is defined in the Supporting Information (Section 3).

If treatment $k^{★}$ is added to the platform trial after the $j_{k}$ stage for treatment k then the correlation equals 0 as there is no shared controls. The proposed methodology allows for different critical boundaries to be used for each treatment k as shown in Equation (2).

If it is assumed that there is an equal number of stages per treatment and equal allocation across all the active treatments, then if one is using the same stopping boundary shape, one can just calculate the FWER. This is because it results in equal PWER for each treatment (Bratton et al. 2016; Choodari‐Oskooei et al. 2020; Greenstreet et al. 2024). This removes the potential issue of time trends with changing allocation ratios. Therefore, to find the boundaries, one can use a single scalar parameter a with the functions $L_{k}=f\left(a\right)$ and $U_{k}=g\left(a\right)$, where g and f are the functions for the shape of the upper and lower boundaries, respectively. This is similar to the method presented in Magirr, Jaki, and Whitehead (2012). If one includes the lower boundaries when calculating the boundaries to control the FWER then these boundaries are binding (Schüler, Kieser, and Rauch 2017). However, for nonbinding boundaries, then Equation (2) can be used, now setting $l_{1},\cdots ,l_{J_{k}-1}$ to be equal to −∞ for all k=1,⋯,K to remove the effect of the lower boundaries. Examples of nonbinding boundaries are given in the Supporting Information (Section 9).

### 2.3 Power

When designing a multi‐arm trial in which all treatments get tested until they are stopped for futility or superiority, regardless of the other treatments, different definitions of power could be considered. The power of a study is focused on the probability that the trial results in some or all of the treatments going forward. The sample size of the study is then found to ensure that the chosen power is greater than or equal to some chosen value, 1−β.

One may be interested in ensuring that at least one treatment is taken forward from the study. This can be split into two types of power discussed in the literature. The first is the disjunctive power (Choodari‐Oskooei et al. 2020; Hamasaki et al. 2021; Urach and Posch 2016) which is the probability of taking at least one treatment forward. The second is the pairwise power which is the probability of taking forward a given treatment (Choodari‐Oskooei et al. 2020; Royston et al. 2011). Pairwise power may be of interest if different interventions are produced by different companies in a platform trial. Therefore, pairwise error control ensures that the power for their given treatment is controlled at the desired level irrespective of any other treatment. In the Supporting Information (Section 4), the equations needed to calculate the disjunctive power ($P_{D}$) are given.

Another way of thinking of powering a study is the probability of taking forward all the treatments which have an effect greater than or equal to the targeted clinically‐relevant effect. This is known as the conjunctive power of a study (Choodari‐Oskooei et al. 2020; Hamasaki et al. 2021; Serra, Mozgunov, and Jaki 2022; Urach and Posch 2016). For the conjunctive power, we prove that it is lowest when all the treatments have the targeted clinically‐relevant effect. Conjunctive power may be of interest in the scenario where new treatments are rare relative to the prevalence of a disease, so the trial is designed to ensure all treatments with a clinically‐relevant effect are found, with high probability.

#### 2.3.1 Pairwise Power

The pairwise power of a treatment is independent of other active treatments. This is because the other active treatments effect has no influence on the treatment of interest as these are independent. Therefore, we only need to consider the probability that the treatment of interest is found superior to the control. The pairwise power for treatment k ($P_{pw,k}$) is

(4) $$P_{pw,k}=\sum_{j_{k}=1}^{J_{k}}\Phi \left(U_{k,j_{k}}^{+}\left(\theta_{k}\right),L_{k,j_{k}}^{+}\left(\theta_{k}\right),{\ddot{\Sigma }}_{k,j_{k}}\right),$$

with

(5) $$\begin{array}{c} \begin{array}{cc} L_{k,j_{k}}^{+}\left(\theta_{k}\right)=(l_{k,1} & -\frac{\theta_{k}}{\sqrt{I_{k,1}}},\cdots ,l_{k,j_{k}-1}-\frac{\theta_{k}}{\sqrt{I_{k,j_{k}-1}}},u_{k,j_{k}}-\frac{\theta_{k}}{\sqrt{I_{k,j_{k}}}}), \end{array} \end{array}$$

(6) $$\begin{array}{cc} U_{k,j_{k}}^{+}\left(\theta_{k}\right)=(u_{k,1} & -\frac{\theta_{k}}{\sqrt{I_{k,1}}},\cdots ,u_{k,j_{k}-1}-\frac{\theta_{k}}{\sqrt{I_{k,j_{k}-1}}},\infty ), \end{array}$$

where $\theta_{k}=\mu_{k}-\mu_{0}$ is the treatment k’s effect compared to the control treatment, and $I_{k,j}=\sigma^{2}\left(n_{k,j}^{-1}+{\left(n_{0,k,j}-n\left(k\right)\right)}^{-1}\right)$. When calculating the required sample size to control the pairwise power, we set $\theta_{k}=\theta^{\prime }$, so we find the power under the targeted clinically relevant effect ($\theta^{\prime }$). This ensures the study is powered at the desired level for a treatment with a clinically relevant effect or greater. The correlation matrix, ${\ddot{\Sigma }}_{k,j_{k}}$, complete correlation structure is

(7) $${\ddot{\Sigma }}_{k,j_{k}}=\left(\begin{array}{cccc} \rho_{\left(k,1),(k,1\right)} & \rho_{\left(k,1),(k,2\right)} & \cdots & \rho_{\left(1,1\right),\left(k,j_{k}\right)}\\ \rho_{\left(k,2),(k,1\right)} & \rho_{\left(k,2),(k,2\right)} & \cdots & \rho_{\left(k,2\right),\left(k,j_{k}\right)}\\ \vdots & \vdots & \ddots & \vdots \\ \rho_{\left(k,j_{k}\right),\left(k,1\right)} & \rho_{\left(k,j_{k}\right),\left(k,2\right)} & \cdots & \rho_{\left(k,j_{k}\right),\left(k,j_{k}\right)} \end{array}\right),$$

where $\rho_{\left(k,j\right),\left(k,j^{★}\right)}$ is defined in the Supporting Information (Section 3).

One can then design the trial so that the pairwise power for each treatment k ($P_{pw,k}$) is greater than or equal to some chosen 1−β for every treatment assuming all treatments have a clinically relevant effect. If one has an equal number of stages per treatment and equal allocation across all the active treatments with the same stopping boundaries, this ensures that pairwise power is equal for each treatment with a clinically relevant effect, so $n_{k}=n_{k^{★}}$ for all $k,k^{★}=1,\cdots ,K$. Therefore, we define $n=n_{k}$ for all k=1,⋯,K. To ensure pairwise power is controlled, keep increasing n until $P_{pw}\geq 1-\beta$, where $P_{pw}=P_{pw,k}$ for all k=1,⋯,K.

If designing a trial in which there is a set number of patients to the control before an active treatment k is added, so n(k) is predefined before calculating the boundaries and sample size, one needs to use an approach such as Algorithm 1. This is because when the sample size increases there is no increase in n(k) for all k. This results in a change in the allocation ratio between r(k) and $r_{0,k,j}$ for each j. Therefore, requiring the bounds to be recalculated for the given r(k). If one focus is on the new arms being added after a set percentage of the way through the trial, this issue no longer persists, as the allocation ratio stays the same so the bounds can be calculated once.

ALGORITHM 1 **Iterative approach to compute the n for the pairwise power with predefined n(K)
**

**Table:** 

0	Begin by assuming n(K)=(0,0,⋯,0) and find the stopping boundaries to control the FWER. Now calculate n such that the pairwise power is greater than or equal to a prespecified (1−β). Then repeat the following iterative steps until the pairwise power, given the true n(K), is greater than (1−β):
1	Find the stopping boundaries to control the FWER for the true predefined n(K) given the current n.
2	Calculate $P_{pw}$ for the given boundaries.
3	If $P_{pw}\geq 1-\beta$ then stop, else increase n by 1 and repeat steps 1–3.

#### 2.3.2 Conjunctive Power

The conjunctive power is defined as the probability of taking forward all the treatments which have a clinically relevant effect. We begin by proving when the conjunctive power is at its lowest. We define the events

$$\begin{array}{cc} B_{k,j}\left(\theta_{k}\right)= & [l_{k,j}+\left(\mu_{k}-\mu_{0}-\theta_{k}\right)I_{k,j}^{1/2}<Z_{k,j}<u_{k,j}+\left(\mu_{k}-\mu_{0}-\theta_{k}\right)I_{k,j}^{1/2}],\\ C_{k,j}\left(\theta_{k}\right)= & [Z_{k,j}>u_{k,j}+\left(\mu_{k}-\mu_{0}-\theta_{k}\right)I_{k,j}^{1/2}], \end{array}$$

where $B_{k,j}\left(\theta_{k}\right)$ defines the event that treatment k continues to the next stage and $C_{k,j}\left(\theta_{k}\right)$ defines the event that treatment k is found superior to the control at stage j. If $\mu_{k}-\mu_{0}=\theta_{k}$ for k=1,⋯,K, the event that $H_{01},\cdots ,H_{0K}$ are all rejected $\left({\bar{W}}_{K}\left(\Theta \right)\right)$ is equivalent to

$$\begin{array}{cc} {\bar{W}}_{K}\left(\Theta \right) & =\cap_{k\in \{1,\cdots ,K\}}(\overset{J_{k}}{\underset{j_{k}=1}{⋃}}[(\cap_{j=1}^{j_{k}-1}B_{k,j}\left(\theta_{k}\right))\cap C_{k,j_{k}}\left(\theta_{k}\right)]), \end{array}$$

where $\Theta =(\theta_{1},\theta_{2},\cdots ,\theta_{K})$ with the convention that $\cap_{i=1}^{0}=\Omega$ where Ω is the whole sample space. We define $\Theta^{\prime }$ as the Θ when $\theta_{k}=\theta^{\prime }$ for all k=1,⋯,K therefore $\Theta^{\prime }=\left(\theta^{\prime },\cdots ,\theta^{\prime }\right)$.

Theorem 2.1 For any Θ, $P\left(\text{reject all}\;H_{0k}\;\text{for which}\;\theta_{k}\geq \theta^{\prime }|\Theta \right)\geq P\left(\text{reject all}\;H_{0k}\;\text{for which}\;\theta_{k}\geq \theta^{\prime }|\Theta^{\prime }\right)$.

The formal proof of Theorem 2.1 is given in the Supporting Information (Section 5). The proof can be split into two key parts. In Part 1, one can show that the conjunctive power, with respect to treatment effect, is a monotonically increasing function. Therefore, the power for treatments with at least a clinically relevant effect is at its lowest when the treatments are at the clinical relevance threshold, $\theta^{\prime }$. Part 2 shows that the probability with respect to the number of intersection hypotheses included is a monotonically decreasing function, so the conjunctive power is smallest when all treatments have a clinically relevant effect.

It follows from Theorem 2.1 that the conjunctive power ($P_{C}$) is minimized when all treatments have the smallest interesting treatment effect. In order to ensure the conjunctive power is greater than level 1−β, we rearrange the events $B_{k,j_{k}}\left(\theta_{k}\right)$ and $C_{k,j}\left(\theta_{k}\right)$ and take advantage of the fact that each event that results in all the treatments being stopped for efficiency are disjoint (Kolmogorov and Bharucha‐Reid 2018) to find

(8) $$P_{C}=P\left({\bar{W}}_{l}\left(\Theta^{\prime }\right)\right)=\sum_{\begin{array}{c} j_{k}=1\\ k=1,2,\text{…},K \end{array}}^{J_{k}}\Phi \left(L_{j_{k}}^{+}\left(\Theta^{\prime }\right),U_{j_{k}}^{+}\left(\Theta^{\prime }\right),\Sigma_{j_{k}}\right),$$

where $U_{j_{k}}^{+}\left(\Theta^{\prime }\right)=\left(U_{1,j_{1}}^{+}\left(\theta^{\prime }\right),\cdots ,U_{K,j_{K}}^{+}\left(\theta^{\prime }\right)\right)$ and $L_{j_{k}}^{+}\left(\Theta^{\prime }\right)=\left(L_{1,j_{1}}^{+}\left(\theta^{\prime }\right),\cdots ,L_{K,j_{K}}^{+}\left(\theta^{\prime }\right)\right)$ with $U_{k,j_{k}}^{+}\left(\theta_{k}\right)$ and $L_{k,j_{k}}^{+}\left(\theta_{k}\right)$ defined in Equations (6) and (5), respectively. The correlation matrix $\Sigma_{j_{k}}$ is the same as that given for FWER in Equation (7).

When one has equal number of stages and fixed allocation ratio to find the sample size, one needs to increase n until $P_{C}\geq 1-\beta$. If one is in the case of fixed n(k) then one can use Algorithm 1, now replacing pairwise power for conjunctive power as once again fixed n(k) results in a change in the allocation ratio between r(k) and $r_{0,k,j}$ for each j.

### 2.4 Sample Size Distribution and Expected Sample Size

The determination of sample size distribution and expected sample size involves calculating the probability for each outcome of the trial, denoted as $Q_{j_{k},q_{k}}$. Here, $q_{k}=\left(q_{1},\cdots ,q_{K}\right)$ is defined, where $q_{k}=0$ indicates that treatment k falls below the lower stopping boundary at point $j_{k}$, and $q_{k}=1$ indicates that treatment k exceeds the upper stopping boundary at point $j_{k}$. We find

$$\begin{array}{cc} Q_{j_{k},q_{k}}= & \Phi ({\tilde{L}}_{j_{k},q_{k}}\left(\Theta \right),{\tilde{U}}_{j_{k},q_{k}}\left(\Theta \right),\Sigma_{j_{k}}), \end{array}$$

with $j_{k}$ one can define ${\tilde{L}}_{j_{k},q_{k}}\left(\Theta \right)=\left({\tilde{L}}_{1,j_{1},q_{1}}\left(\theta_{1}\right),\cdots ,{\tilde{L}}_{K,j_{K},q_{K}}\left(\theta_{K}\right)\right)$ and ${\tilde{U}}_{j_{k},q_{k}}\left(\Theta \right)=({\tilde{U}}_{1,j_{1},q_{1}}\left(\theta_{1}\right)$, $\cdots ,{\tilde{U}}_{K,j_{K},q_{K}}\left(\theta_{K}\right))$ where

$$\begin{array}{ccc} {\tilde{L}}_{k,j_{k},q_{k}}\left(\theta_{k}\right) & = & (l_{k,1}-\frac{\theta_{k}}{\sqrt{I_{k,1}}},\cdots ,l_{k,j_{k}-1}-\frac{\theta_{k}}{\sqrt{I_{k,j_{k}-1}}},\\ & & \left[1\left(q_{k}=0\right)\left(-\infty \right)+u_{k,j_{k}}\right]-\frac{\theta_{k}}{\sqrt{I_{k,j_{k}}}}),\\ {\tilde{U}}_{k,j_{k},q_{k}}\left(\theta_{k}\right) & = & (u_{k,1}-\frac{\theta_{k}}{\sqrt{I_{k,1}}},\cdots ,u_{k,j_{k}-1}-\frac{\theta_{k}}{\sqrt{I_{k,j_{k}-1}}},\\ & & \left[1\left(q_{k}=1\right)\left(\infty \right)+l_{k,j_{k}}\right]-\frac{\theta_{k}}{\sqrt{I_{k,j_{k}}}}), \end{array}$$

respectively. The correlation matrix $\Sigma_{j_{k}}$ is given in Equation (7). The $Q_{j_{k},q_{k}}$ are associated with their given total sample size $N_{j_{k},q_{k}}$ for that given $j_{k}$ and $q_{k}$

$$\begin{array}{cc} N_{j_{k},q_{k}}= & (\sum_{k=1}^{K}n_{k,j_{k}})+\max_{k\in 1,\cdots K}\left(n_{0,k,j_{k}}\right). \end{array}$$

This shows that the control treatment continues being recruited to until, at the earliest, the last active treatment to be added has had at least one analysis. To obtain the sample size distribution, as similarly done in Greenstreet et al. (2024), we group all the values of $j_{k}$ and $q_{k}$ that gives the same value of $N_{j_{k},q_{k}}$ with its corresponding $Q_{j_{k},q_{k}}$. This set of $Q_{j_{k},q_{k}}$ is then summed together to give the probability of the realization of this sample size. To calculate the sample size distribution for each active arm, group $n_{k,j_{k}}$ with its corresponding $Q_{j_{k},q_{k}}$ and this can similarly be done for the control treatment. The expected sample size for a given Θ, denoted as E(N|Θ), is obtained by summing all possible combinations of $j_{k}$ and $q_{k}$,

(9) $$E\left(N|\Theta \right)=\sum_{\begin{array}{c} j_{k}=1\\ k=1,2,\text{…},K \end{array}}^{J_{k}}\sum_{\begin{array}{c} q_{k}\in \left\{0,1\right\}\\ k=1,2,\text{…},K \end{array}}Q_{j_{k},q_{k}}N_{j_{k},q_{k}}.$$

The expected sample size for multiple different treatment effects ($\Theta =(\theta_{1},\cdots ,\theta_{K})$) can then be found using Equation (9).

## 3 Motivating Trial Example

### 3.1 Setting

One example of a platform trial is FLAIR, which focused on chronic lymphocyte leukemia (Howard et al. 2021). FLAIR initially planned to incorporate an additional active treatment arm and conduct an interim analysis midway through the intended sample size for each treatment. During the actual trial, two extra arms were introduced, including an additional control arm. The original trial design primarily addressed the pairwise type I error due to the inclusion of both additional experimental and control arms.

Following Greenstreet et al. (2024), a hypothetical trial that mirrors some aspects of FLAIR will be studied. In this hypothetical trial, the FWER in the strong sense will be controlled. Controlling the FWER may be seen as crucial in this scenario, as the trial aims to assess various combinations of treatments involving a common compound for all active treatments (Wason, Stecher, and Mander 2014). There is an initial active treatment arm, a control arm, and a planned addition of one more active treatment arm during the trial. We apply the proposed methodology to ensure FWER control and consider the conjunctive power and pairwise power.

The pairwise power is the main focus of the simulation study rather than the disjunctive power, as a potential drawback of disjunctive power is it is highly dependent on the treatment effect of all the treatments in the study, even the ones with a small or even negative treatment effect. For example, assume one treatment has a clinically relevant effect and the rest have an effect equal to the control treatment, then the disjunctive power will keep increasing the more treatments that are added if one keeps the same bounds, even though the probability of taking the correct treatment forward does not increase. Equally, the minimum the disjunctive power can be is equal to the pairwise power. This is when only one treatment has a clinically relevant effect and the rest have an extreme negative effect. A further advantage of the pairwise power is it gives the probability of the treatment with the greatest treatment effect being found.

Considering the planned effect size from FLAIR, we assume an interesting treatment difference of $\theta^{\prime }=-\log\left(0.69\right)$ and a standard deviation of σ=1. It should be noted that while FLAIR used a time‐to‐event endpoint with 0.69 representing the clinically relevant hazard ratio between the experimental and control groups, our hypothetical trial will focus on continuous endpoints using a normal approximation of time‐to‐event endpoints as discussed in Jaki and Magirr (2013). The desired power is 80%. We will maintain the same power level as FLAIR while targeting a one‐sided FWER of 2.5%. The active treatment arms interim analysis will be conducted midway through its recruitment and 1:1 allocation will be used between the control and the active treatments as done in FLAIR (Hillmen et al. 2023).

The difference between a design which controls the pairwise power and the conjunctive power will be studied in Section 3.2. In Sections 3.3 and 3.4, the effect of different numbers of patients recruited to the control before the second treatment is added (n(2)) will be studied with the focus being on expected sample size and maximum sample size of the trial. The designs will be compared to running two completely separate independent trials for each of the two active treatments. In Section 3.5, the effect of using a more liberal FWER control compared to type I error control for the separate trials is studied for trials with three and four active arms.

### 3.2 Comparing the Two Types of Power

We will consider the effect of adding the second treatment halfway through recruitment of the first active treatment, both for ensuring pairwise power and conjunctive power are at 80%. Binding triangular stopping boundaries will be used (Li, Herrmann, and Rauch 2020; Wason and Jaki 2012; Whitehead 1997) with the nonbinding triangular stopping boundaries given in the Supporting Information (Section 9). The stopping boundaries are the same regardless of if one is controlling pairwise power or conjunctive power as $r\left(2\right)=r_{1,1}$ for both. The stopping boundaries are given in Table 1 and are equal for both designs. The calculations were carried out using R (R Core Team 2021) with the method given here having the multivariate normal probabilities being calculated using the packages mvtnorm (Genz et al. 2021) and gtools (Warnes et al. 2021). Code is available at https://github.com/pgreenstreet/Platform_trial_multiple_superior.

**TABLE 1:** The stopping boundaries and sample size of the proposed designs, for control of both pairwise power and of conjunctive power.

Design controlling	$\left(\begin{array}{c} U_{1}\\ U_{2} \end{array}\right)$	$\left(\begin{array}{c} L_{1}\\ L_{2} \end{array}\right)$	$\left(\begin{array}{cc} n_{1,1} & n_{1,2}\\ n_{2,1} & n_{2,2} \end{array}\right)$	$\left(\begin{array}{cc} n_{0,1,1} & n_{0,1,2}\\ n_{0,2,1} & n_{0,2,2} \end{array}\right)$	$\left(\begin{array}{c} n(1)\\ n(2) \end{array}\right)$	max(N)
Pairwise power	$\left(\begin{array}{cc} 2.501 & 2.358\\ 2.501 & 2.358 \end{array}\right)$	$\left(\begin{array}{cc} 0.834 & 2.358\\ 0.834 & 2.358 \end{array}\right)$	$\left(\begin{array}{cc} 76 & 152\\ 76 & 152 \end{array}\right)$	$\left(\begin{array}{cc} 76 & 152\\ 152 & 228 \end{array}\right)$	$\left(\begin{array}{c} 0\\ 76 \end{array}\right)$	532
Conjunctive power	$\left(\begin{array}{cc} 2.501 & 2.358\\ 2.501 & 2.358 \end{array}\right)$	$\left(\begin{array}{cc} 0.834 & 2.358\\ 0.834 & 2.358 \end{array}\right)$	$\left(\begin{array}{cc} 96 & 192\\ 96 & 192 \end{array}\right)$	$\left(\begin{array}{cc} 96 & 192\\ 192 & 288 \end{array}\right)$	$\left(\begin{array}{c} 0\\ 96 \end{array}\right)$	672

Based on the two designs in Table 1, the conjunctive power, pairwise power, and disjunctive power for different values of $\theta_{1}$ and $\theta_{2}$ alongside the expected sample size are given in Table 2. The values of $\theta_{1}$ and $\theta_{2}$ are chosen to study the effects under the global null hypothesis, when treatments have a clinically relevant effect and when one of the active treatments performs considerably worse than the rest.

**TABLE 2:** Operating characteristics of the proposed designs under different values of $\theta_{1}$ and $\theta_{2}$, for control of both pairwise power and of conjunctive power.

**Design for pairwise power**						
Treatment effect		Pairwise power		Conjunctive power	Disjunctive power	Expected sample size
$\theta_{1}$	$\theta_{2}$	$P_{PW,1}$	$P_{PW,2}$	$P_{C}$	$P_{D}$	$E(N|\theta_{1},\theta_{2})$
$\theta^{\prime }$	$\theta^{\prime }$	0.800	0.800	0.660	0.941	420.6
$\theta^{\prime }$	0	0.800	0.013	0.800	0.802	372.7
$\theta^{\prime }$	−∞	0.800	0	0.800	0.800	342.9
0	$\theta^{\prime }$	0.013	0.800	0.800	0.802	396.6
0	0	0.013	0.013	1	0.025	348.7
−∞	$\theta^{\prime }$	0	0.800	0.800	0.800	381.7

**Design for conjunctive power**						
Treatment effect		Pairwise power		Conjunctive power	Disjunctive power	Expected sample size
$\theta_{1}$	$\theta_{2}$	$P_{PW,1}$	$P_{PW,2}$	$P_{C}$	$P_{D}$	$E(N|\theta_{1},\theta_{2})$
$\theta^{\prime }$	$\theta^{\prime }$	0.890	0.890	0.801	0.979	508.1
$\theta^{\prime }$	0	0.890	0.013	0.890	0.890	463.0
$\theta^{\prime }$	−∞	0.890	0	0.890	0.890	425.4
0	$\theta^{\prime }$	0.013	0.890	0.890	0.891	485.6
0	0	0.013	0.013	1	0.025	440.5
−∞	$\theta^{\prime }$	0	0.890	0.890	0.890	466.7

In Table 2, it can be seen that the pairwise power for the treatment with a clinically relevant effect is equal to the disjunctive power when the other treatment has an extremely negative treatment effect compared to the control. This is because there is no longer a chance that the other treatment can be taken forward. Therefore, $\theta_{1}=-\infty$, $\theta_{2}=\theta^{\prime }$ or $\theta_{1}=\theta^{\prime }$, $\theta_{2}=-\infty$, is the point when the pairwise, disjunctive, and conjunctive power are all equal. In Table 2, it is shown that when both treatments have effect 0 the disjunctive power is equal to the FWER for the trial. In addition, when a treatment has effect 0 this results in the pairwise power for that treatment equaling the PWER. Furthermore, Table 2 shows when there is only one treatment with a clinically relevant effect, the conjunctive power equals the pairwise power of that treatment. When neither treatment has a clinically relevant effect the conjunctive power equals 100%, as there are no treatments with a clinically relevant effect that need to be found.

The results for using both O'Brien and Fleming (O'Brien and Fleming 1979) and Pocock boundaries (Pocock 1977) are shown, with the futility boundary equal to 0 (Magirr, Jaki, and Whitehead 2012), for both binding and nonbinding futility boundaries, in the Supporting Information (Sections 8 and 9). Overall, Tables 1 and 2 have shown that the choice of type of power to control may be highly dependent on the sample size available, as if the design ensures conjunctive power of level 1−β it will ensure pairwise power of at least 1−β but the opposite does not hold. However, the sample size for a trial designed for pairwise power will be less than that of a design for conjunctive power.

### 3.3 Comparison With Running Separate Trials

This section studies the effect on maximum and expected sample size depending on when the additional treatment arm is added to the platform trial. The examples for both conjunctive power and pairwise power are compared to running two separate trials. There are two settings for separate trials which are considered. Setting 1 is when the type I error across both the trials is set to be 2.5%, therefore, the type I error for each is $1-\sqrt{1-0.025}=1.26\%$. For Setting 2, the type I error of each trial is controlled at 2.5%. For the separate trials which are compared to the pairwise power, the power level for each is set to 80%. This results in the following sample size and stopping boundaries for the two trials for Setting 1,

$$\begin{array}{ccc} U_{1} & = & \left(\begin{array}{cc} 2.508 & 2.364 \end{array}\right),\;\;\;\;L_{1}=\left(\begin{array}{cc} 0.836 & 2.364 \end{array}\right),\\ & & \left(\begin{array}{cc} n_{1,1} & n_{1,2} \end{array}\right)=\left(\begin{array}{cc} 77 & 154 \end{array}\right), \end{array}$$

with $n_{0,1,1}=n_{1,1}$, $n_{0,1,2}=n_{1,2}$, and n(1)=0. Setting 2 gives:

$$\begin{array}{ccc} U_{1} & = & \left(\begin{array}{cc} 2.222 & 2.095 \end{array}\right),\;\;\;\;L_{1}=\left(\begin{array}{cc} 0.741 & 2.095 \end{array}\right),\\ & & \left(\begin{array}{cc} n_{1,1} & n_{1,2} \end{array}\right)=\left(\begin{array}{cc} 65 & 130 \end{array}\right), \end{array}$$

with $n_{0,1,1}=n_{1,1}$, $n_{0,1,2}=n_{1,2}$, and n(1)=0. For comparison with the conjunctive power designs, the probability of finding both treatments across the two trials is set to 80%. The required power for each trial is therefore $\sqrt{1-\beta }=0.894$. The boundaries remain the same for both settings as the type I error remains the same. The new sample size for Setting 1 is $\left(\begin{array}{cc} n_{1,1} & n_{1,2} \end{array}\right)=\left(\begin{array}{cc} 98 & 196 \end{array}\right)$ and for Setting 2 is $\left(\begin{array}{cc} n_{1,1} & n_{1,2} \end{array}\right)=\left(\begin{array}{cc} 85 & 170 \end{array}\right)$.

**FIGURE 1:**
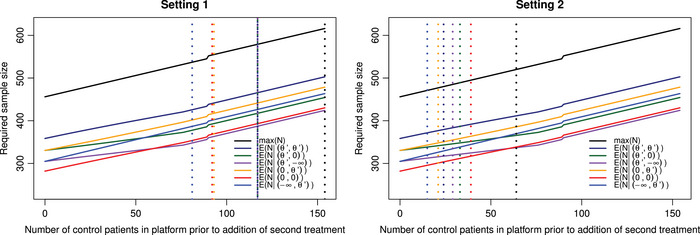
Both panels give the maximum sample size and the expected sample size under different $\theta_{1},\theta_{2}$ depending on the value n(2), for the pairwise power control of 80%. Left panel: dashed vertical lines correspond to the points where the maximum/expected sample size of the trial is now greater than running two separate trials with type I error control across both trials set to 2.5%. The lines $\theta_{1}=\theta^{\prime }$, $\theta_{2}=\theta^{\prime }$; $\theta_{1}=\theta^{\prime }$, $\theta_{2}=0$; and $\theta_{1}=\theta^{\prime }$, $\theta_{2}=-\infty$ are at n(2)=117 for all three configurations. Right panel: dashed vertical lines correspond to the points where the maximum/expected sample size of the trial is now greater than running two separate trials with type I error control for each trial set to 2.5%.

**FIGURE 2:**
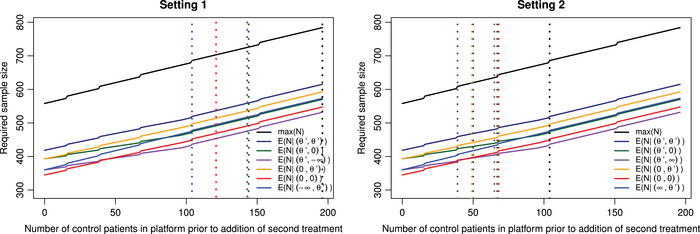
The maximum sample size and the expected sample size under different $\theta_{1},\theta_{2}$ depending on the value n(2), for the conjunctive power control of 80%. Left panel: dashed vertical lines correspond to the points where the maximum/expected sample size of the trial is now greater than running two separate trials under Setting 1. The lines $\theta_{1}=\theta^{\prime }$, $\theta_{2}=\theta^{\prime }$ and $\theta_{1}=\theta^{\prime }$, $\theta_{2}=-\infty$ are at n(2)=143 for both configurations; $\theta_{1}=\theta^{\prime }$, $\theta_{2}=0$ and $\theta_{1}=0$, $\theta_{2}=0$ are at n(2)=121 for both configurations. Right panel: dashed vertical lines correspond to the points where the maximum/expected sample size of the trial is now greater than running two separate trials under Setting 2.

Figure 1 gives the maximum sample size and the expected sample size under different $\theta_{1},\theta_{2}$ depending on when the second treatment is added, for the pairwise power control of 80%. Figure 2 gives similar results however the focus now is on control of the conjunctive power at 80%.

As indicated in Figure 1, when controlling the pairwise power, if the second active treatment is introduced at the beginning of the trial, the total sample size required is 456, whereas if it is added at the end of recruitment for treatment 1, the total sample size becomes 616. This increase in sample size is attributable to two factors. First, there is a necessity to increase the number of patients recruited to the control group until treatment 2 has completed the trial. Second, the decrease in correlation between the two treatments results in an enlargement of the boundaries to maintain control over the FWER. It is this secondary factor which causes the small jumps in maximum sample size seen in Figures 1 and 2.

In Figure 1, when comparing the platform designs with pairwise power control to running two separate trials, it can be seen that, for the case where the pairwise error for each trial is 2.5%, once the second treatment is added after 64 patients have been recruited to the control (n(2)≥64), the maximum sample size of running the platform design is greater than or equal to that of running two separate trials, which is 520 patients. However, when controlling the error across both separate trials, the maximum sample size is now the same as when adding the second treatment at the end of recruitment for the first treatment in the platform design so 616. For Setting 1, it can be seen that the expected sample size for separate trials can be better than that of the platform design. In the case of $\theta_{1}=-\infty$ and $\theta_{2}=\theta^{\prime }$, then once n(2)≥81, the expected sample size of running the platform design is greater than that of running two separate trials. For Setting 1, the lines for $\theta_{1}=\theta^{\prime }$, $\theta_{2}=\theta^{\prime }$; $\theta_{1}=\theta^{\prime }$, $\theta_{2}=0$; and $\theta_{1}=\theta^{\prime }$, $\theta_{2}=-\infty$ are all at the point n(2)=117. When studying the expected sample size of Setting 2 compared to the platform designs, it can be seen that if $\theta_{1}=-\infty$ and $\theta_{2}=\theta^{\prime }$ then once n(2)≥15, the expected sample size of running the platform design is greater than that of running two separate trials. The expected sample size for two separate trials when $\theta_{1}=-\infty$ and $\theta_{2}=\theta^{\prime }$ is 319.5.

In Figure 2, the equivalent results to Figure 1 when controlling the conjunctive power are shown, with the maximum sample size now ranging from 558 if the second active treatment is introduced at the beginning of the trial, to 784 if it is added at the end of recruitment for treatment 1. For Setting 1 in Figure 2, n(2)=143 is the point for both $\theta_{1}=\theta^{\prime }$, $\theta_{2}=\theta^{\prime }$ and $\theta_{1}=\theta^{\prime }$, $\theta_{2}=-\infty$, also n(2)=121 is the point for both $\theta_{1}=0$, $\theta_{2}=\theta^{\prime }$ and $\theta_{1}=0$, $\theta_{2}=0$.

It is worth noting that there is the underlying assumption that in the platform trial there will be no pause in patient recruitment even if for a period there is only the control treatment. The case of continuous recruitment can be seen as a worst‐case scenario as there are multiple practical issues around pausing recruitment and the time this may take (Constable et al. 2020; Mitchell et al. 2020). However, in Section 3.4, the results when one does pause recruitment during periods of no active treatment are shown.

Overall, Figures 1 and 2 have shown there maybe times that there is no benefit to running a platform trial with regard to sample size, depending on when the later treatment is added to the trial. This issue is further emphasized when there is not the expectation to control the type I error across all the individual trials as seen in Setting 2. The Supporting Information (Section 7) provides a table which gives the maximum sample size of the trial for multiple values of n(2) based on Figures 1 and 2.

### 3.4 Comparison With Running Separate Trials When Allowing for Pauses in Recruitment

**FIGURE 3:**
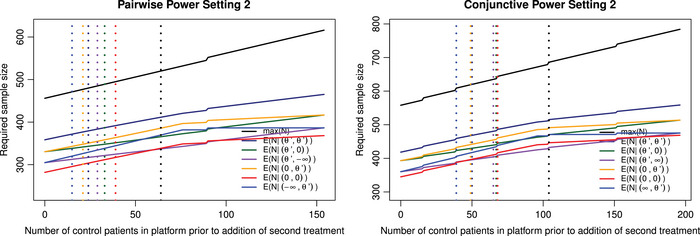
The maximum sample size and the expected sample size under different $\theta_{1},\theta_{2}$ depending on the value n(2) when allowing for pauses in recruitment, with the dashed vertical lines corresponding to the points where the maximum/expected sample size of the trial is now greater than running two separate trials under Setting 2. Left panel: The pairwise power is at 80%. Right panel: The conjunctive power is at 80%.

Figure 3 gives the maximum sample size and the expected sample size under different $\theta_{1},\theta_{2}$ depending on when the second treatment is added, for the pairwise power of 80% and conjunctive power of 80%, respectively, when allowing for pauses in recruitment. In the Supporting information (Section 6), the equation to calculate the expected sample size when allowing pauses in recruitment is given. In Figure 3, it can now be seen that the rate at which the expected sample size increases slows with increased time before the second treatment is added after the first interim analyses. This is because there is a chance that active treatment 1 will be stopped at the first stage. Therefore, there will be a period of paused recruitment. How extreme the decrease in rate of the expected sample size increase depends on the configuration studied. For example, if active treatment 1 has a very negative effect then it will almost always stop recruitment at the first stage, so resulting in a very extreme decrease in the rate of expected sample size increase.

For Setting 1, the reason there are points in Figures 1 and 2 in which the platform trial performs worse than running two separate trials is because there is the underlying assumption that in the platform trial, there will be no pause in patient recruitment even if for a period there is only the control treatment. It is worth noting that if both the platform design and two separate trials are required to control the FWER then the platform approach is never worse than running two trials when pausing recruitment. They are equal at the point when the second treatment is added after the maximum planned sample size for treatment 1, so for pairwise power this is when n(2)=154 and max(N)=616; for conjunctive power this is when n(2)=196 and max(N)=784.

The dashed vertical lines on Figure 3 correspond to the points where the maximum/expected sample size of the trial is now greater than running two separate trials under Setting 2. The ability to pause the trial in this case has no effect on the value of n(2) for which the separate trials' expected sample size is less than that of running the platform for the configurations studied. This is because $n\left(2\right)<n_{0,1,1}$ for the configurations studied so at these points the trial will never pause recruitment.

### 3.5 Comparison With Running Separate Trials Under Different Controls of Type I Error

When designing a multi‐arm trial, one may find that the expected control of the FWER is less than that of the type I error control for an individual trial, as seen in the TAILoR trial for example (Pushpakom et al. 2020, 2015). Therefore, in Table 3, we consider the effect of allowing FWER control of 5% one‐sided compared to 2.5% type I error for the individual trials. In this table, the same design parameters were used as above; however, now the number of active arms has increased in the hypothetical trial to 3 or 4, and the number of stages is now either 1, 2, or 3. In Table 3, the focus is on controlling the power at the desired 80% level with the pairwise power being the focus for the top half and conjunctive power for the bottom half. When controlling the conjunctive power, the power for each separate trial is ${\left(1-\beta \right)}^{1/k}$. In these hypothetical trials, it is assumed that each one of the arms is added sequentially, with an equal gap between each one. Therefore, in the three active arm cases if the second arm is added after 20 patients have been recruited to the control then the third arm will be added after a total of 40 patients have been recruited to the control.

**TABLE 3:** The comparison of using the proposed platform design with FWER of 5% one‐sided against running separate trials with type I error control of each at 2.5% one‐sided, for different numbers of arms and stages. The sample size for the platform trial designs ranges from all the treatments starting at once (n(k)=0 for k=1,⋯,K), to each treatment not starting until the maximum number of patients are recruited to the previous treatment ($n\left(k\right)=n_{0,k-1,J}$ for all k=2,⋯,K).

**Design for pairwise power**										
Active arms	Stages	Separate trial		Platform trial range		$\min_{n(2)}(\max\left(N_{s}\right)$	$\min_{n(2)}\left(E\left(N_{s}|\Theta \right)\leq E\left(N|\Theta \right)\right)$			
K	J	n	$\max(N_{s})$	n	max(N)	≤max(N))	$\Theta_{1}$	$\Theta_{2}$	$\Theta_{3}$	$\Theta_{4}$
3	1	115	690	123–128	492–768	90	90	90	90	90
3	2	65	780	69–72	552–864	105	73	72	62	66
3	3	46	828	49–50	588–900	114	68	67	55	60
4	1	115	920	131–138	655–1104	79	79	79	79	79
4	2	65	1040	73–76	730–1216	94	61	62	54	59
4	3	46	1104	51–53	765–1272	103	59	58	49	55

**Design for conjunctive power**										
Active arms	Stages	Separate trial		Platform trial range		$\min_{n(2)}(\max\left(N_{s}\right)$	$\min_{n(2)}\left(E\left(N_{s}|\Theta \right)\leq E\left(N|\Theta \right)\right)$			
K	J	$n_{1,1}$	$\max(N_{s})$	$n_{1,1}$	max(N)	≤max(N))	$\Theta_{1}$	$\Theta_{2}$	$\Theta_{3}$	$\Theta_{4}$
3	1	171	1026	168–187	672–1122	143	143	143	143	143
3	2	97	1164	95–105	760–1260	166	107	109	101	106
3	3	68	1224	67–73	804–1314	174	98	99	92	98
4	1	185	1480	190–215	950–1720	140	140	140	140	140
4	2	105	1680	107–119	1070–1904	167	102	109	103	109
4	3	74	1776	74–83	1110–1992	182	93	99	93	99

*Note:* $N_{s}$ is the sample size of running K separate trials, $\Theta_{1}$: $\theta_{k}=\theta^{\prime }$ for k=1,⋯,K; $\Theta_{2}$: $\theta_{1}=\theta^{\prime }$, $\theta_{k}=0$ for k=2,⋯,K; $\Theta_{3}$: $\theta_{K}=\theta^{\prime }$, $\theta_{k}=0$ for k=1,⋯,K−1; $\Theta_{4}$: $\theta_{k}=0$ for k=1,⋯,K.

In Table 3, the first two columns give the number of active arms and stages for the platform trial, respectively. The third and fourth columns then give the sample size per stage and the maximum sample size of the individual trials, respectively. The next two columns give the range in sample size per stage and maximum sample size for the platform trial design. The platform trial designs range from all the treatments starting at once (n(k)=0 for k=1,⋯,K), to each treatment not starting until the maximum number of patients are recruited to the previous treatment ($n\left(k\right)=n_{0,k-1,J}$ for all k=2,⋯,K). The remaining columns give when there is no benefit with regards to the maximum and expected sample size of conducting a platform trial compared to running separate trials, with respect n(k)−n(k−1). The value of n(k)−n(k−1)=n(2) as the first treatment is added at the beginning of the trial. In the Supporting Information (Section 10), the plots for the two‐stage and three‐stage example trials as given in Table 3 are shown.

Using Table 3, for the three active arms, two‐stage example each separate trial has $n_{1,1}=65$ and $n_{1,2}=130$. The total maximum sample size of running these three separate trials is therefore 780. The platform trial's maximum sample size ranges from 552 if all the treatments begin at once to 864 if $n\left(k\right)=n_{0,k-1,J}$ for all k=2,⋯,K. Once the second treatment is planned to be added after 105 patients recruited to the control (therefore 210 recruited to the control before treatment 3), there is no benefit in using the platform design with respect to maximum sample size. For the expected sample size, four different configurations of the treatment effects are studied. The first ($\Theta_{1}$) assumes all the treatments have the clinically relevant effect, so $\theta_{k}=\theta^{\prime }$ for k=1,⋯,K. The second ($\Theta_{2}$) assumes only the first treatment has a clinically relevant effect and the rest have an effect equal to that of the control treatment, so $\theta_{1}=\theta^{\prime }$, $\theta_{k}=0$ for k=2,⋯,K. The third ($\Theta_{3}$) assumes only the last treatment has a clinically relevant effect and the rest equal the control, so $\theta_{K}=\theta^{\prime }$, $\theta_{k}=0$ for k=1,⋯,K−1. The fourth configuration ($\Theta_{4}$) assumes all the treatments have an effect equal to that of the control treatment, so the global null hypothesis, so $\theta_{k}=0$ for k=1,⋯,K. For the expected sample size for the four treatment effect configurations studied here, there is no benefit in using a platform trial after potentially just 62 patients if $\Theta_{3}$ is true, this does rise to 73 if $\Theta_{1}$ is true, if the focus is on sample size.

This section has shown that there are periods in which using a platform trial can be beneficial with regards to sample size if one can use a more liberal type I error control compared to that used for individual trials. However, this has also shown that if treatments are added late into the trial there may not be benefit, so highlighting the importance of considering which trial design should be use.

## 4 Discussion

This paper has built on the work of Greenstreet et al. (2024) to show how one can control the FWER for a trial in which the treatments can be preplanned to be added at any point. This work has then studied the different approaches for powering the trial in which the trial will continue even if a superior treatment is found. This paper shows how the expected sample size and sample size distribution can be found. Finally, a hypothetical trial, motivated by FLAIR (Howard et al. 2021) is discussed. Section 3.2 evaluates the pairwise and conjunctive power when the second active treatment is added halfway through recruitment for the first active treatment. We investigate the operating characteristics for multiple values of $\theta_{1}$ and $\theta_{2}$. Then the section goes on to study the effect of adding the later treatments at different points in the platform design and compares these trial designs to running separate trials.

The designs' flexibility to incorporate the addition of treatments at any point during a trial allows for the creation of multiple designs at the development stage of the trial that depend on when and how many treatments are introduced. As a result, one can present multiple options and then use the trial design, which is expected to match the reality of the trial closest. If in reality there are less treatments added than planned, the control of FWER rate will be maintained. Due to the bounds being designed to control the FWER across all the hypotheses, not adding a treatment and so removing a hypothesis reduces the maximum value of the FWER. If, however, more arms are added than were considered at the design stage, or at later time points, there is no longer guaranteed control of the trials' errors. However, one could instead adjust the trial using an unplanned approach (Burnett, König, and Jaki 2024). However, when using an unplanned approach, one should be aware of the potential issues. These include that both current and later treatments can become underpowered due to the limited amount of resources for the trial. If one wants to reduce this, then further funding is needed to allow for the additional patients required. Further to this, it is very difficult, and in some cases impossible, to ensure that the type I error is evenly shared across all the treatments. In addition, as argued by Posch and Proschan (2012), unplanned adaptations will always question the confirmatory nature of a clinical trial. Therefore, Posch and Proschan (2012) argue unplanned adaptations should be considered only when deemed absolutely necessary.

This paper shows the large influence the choice of power in a platform trial can have on the required sample size and operating characteristics. In the scenario where recruitment can be easily done, then one should consider designing the trial to control the conjunctive power to, with high probability, find all the treatments with a clinically relevant effect. This allows clinicians to be more informed about which treatments have a desirable effect. The choice of which treatment to give can then be made based on other properties of the treatments, such as its side effects. In many trials, the number of patients can be a very limiting factor with a very high cost of recruiting patients. In this case, there may be a drive to cut sample size and a desire to find any treatment that has a positive treatment effect. Therefore, the pairwise power or disjunctive power should be used. As shown in Section 3.2, the disjunctive power's lowest value is the pairwise power, so as the treatment effects of the active treatments are not known before the trial, we would recommend using the pairwise power, ensuring control of both pairwise power and disjunctive power.

Based on the hypothetical trial, the effects of continuous recruitment, or allowing recruitment to be paused during periods of no active treatments, are studied in Sections 3.3 and 3.4, respectively. For the hypothetical trial studied, there is no effect on when running separate trials outperforms the platform approach with respect to sample size, whether one pauses recruitment or not, if the separate trials do not control FWER. However, when both designs control the FWER, then it was shown there is always benefit in using a platform approach compared to separate trials when pausing is possible. In addition, in the Supporting Information (Section 6), a method for calculating the expected sample size when allowing for pauses in recruitment is given, which builds on Equation (9). Both allowing and not allowing for pauses in recruitment have been considered as continuous recruitment can be seen as a worst‐case scenario and there can be multiple practical issues around pausing a trial as discussed in Constable et al. (2020) and Mitchell et al. (2020). However, pausing the recruitment immediately can be seen as a best‐case scenario so is also of interest.

This paper has therefore highlighted a potential issue of increased expected and maximum sample size when requiring strong control of FWER for a platform trial in which an arm is added later. If one would run two completely separate trials, the FWER control across the trials would likely not be expected. From the perspective of type I error control, the comparison is not equivalent, however, in reality this is likely an issue faced when choosing if to design a platform trial, or not. As a result, there is a lot of time where there is no benefit to the platform trial design with regards to maximum or expected sample size as was shown in Figures 1, 2, 3 for Setting 2. This work reiterates the importance of the discussions around type I error control in platform trials (Howard et al. 2018; Molloy et al. 2022; Nguyen, Hees, and Hofner 2023; Proschan and Follmann 1995; Proschan and Waclawiw 2000; Wason et al. 2016; Wason, Stecher, and Mander 2014).

If one instead wants to control the pairwise error, as done, for example, in STAMPEDE (Sydes et al. 2009), one can use Equation (4), now setting $\theta_{k}=0$ for all k=1,⋯,K. An additional advantage of using the PWER, if controlling the pairwise power, is that the stopping boundaries and the sample size required for each active arm are independent of when the arm is added. Therefore, the only change will be how many patients need to be recruited to the control. However, one may find PWER in a platform trial insufficient for error control (Molloy et al. 2022; Wason, Stecher, and Mander 2014) and may not meet the regulators requirements.

Building upon this research, a study could be conducted to investigate the impact of having different numbers of stages and stopping boundaries while maintaining equal power and type I error for each treatment, utilizing the approach described in Section 2.1. However, such an investigation would likely require multiple changes in the allocation ratio, resulting in potential issues with time trends. One could therefore examine methods to handle these time trends, as explored in Greenstreet et al. (2024), Lee and Wason (2020), Marschner and Schou (2022), and Roig et al. (2023). Furthermore, a change in allocation ratio between treatments can result in different PWER and pairwise power for each treatment if using the same boundaries for each treatment, so one could use an iterative approach such as that discussed in Greenstreet et al. (2024). Equally, one could study the effect of using nonconcurrent controls, but once again this can face a large issue with time trends and the resulting bias. However, one could look into incorporating approaches to reduce the bias potentially caused (Lee and Wason 2020; Marschner and Schou 2022; Saville et al. 2022; Wang et al. 2022).

This paper has given a general formulation for designing a preplanned platform trial with a normal continuous endpoint, and using the work of Jaki and Magirr (2013) one could apply this methodology to other endpoint such as time‐to‐event used in FLAIR (Howard et al. 2021). When using this approach, one should be aware of computational issues from calculating high‐dimensional multivariate normal distributions, if one has a large number of arms and stages in the trial design. If this is an issue, then one can restrict to only adding arms at the interims so one can utlize the method of Dunnett (1955) as discussed in Greenstreet et al. (2024) and Magirr, Jaki, and Whitehead (2012).

## Conflicts of Interest

Alun Bedding is a shareholder of Roche Products Ltd. The other authors declare no conflicts of interest.

### OPEN RESEARCH BADGES

This article has earned an Open Data badge for making publicly available the digitally‐shareable data necessary to reproduce the reported results. The data is available in the Supporting Information section.

This article has earned an open data badge “**Reproducible Research**” for making publicly available the code necessary to reproduce the reported results. The results reported in this article could fully be reproduced.

## Supporting information

Supporting Information

Supporting Information

## Data Availability

The data that support the findings of this study are openly available in Platform_trial_multiple_superior at https://github.com/pgreenstreet/Platform_trial_multiple_superior.
