# Supplementary figures and images for: A Preplanned Multi‐Stage Platform Trial for Discovering Multiple Superior Treatments With Control of FWER and Power
Source: Biom J. 2024 Dec 22;67(1):e70025. doi: 10.1002/bimj.70025 (PMC11664203; doi:10.1002/bimj.70025)

## Setting 2

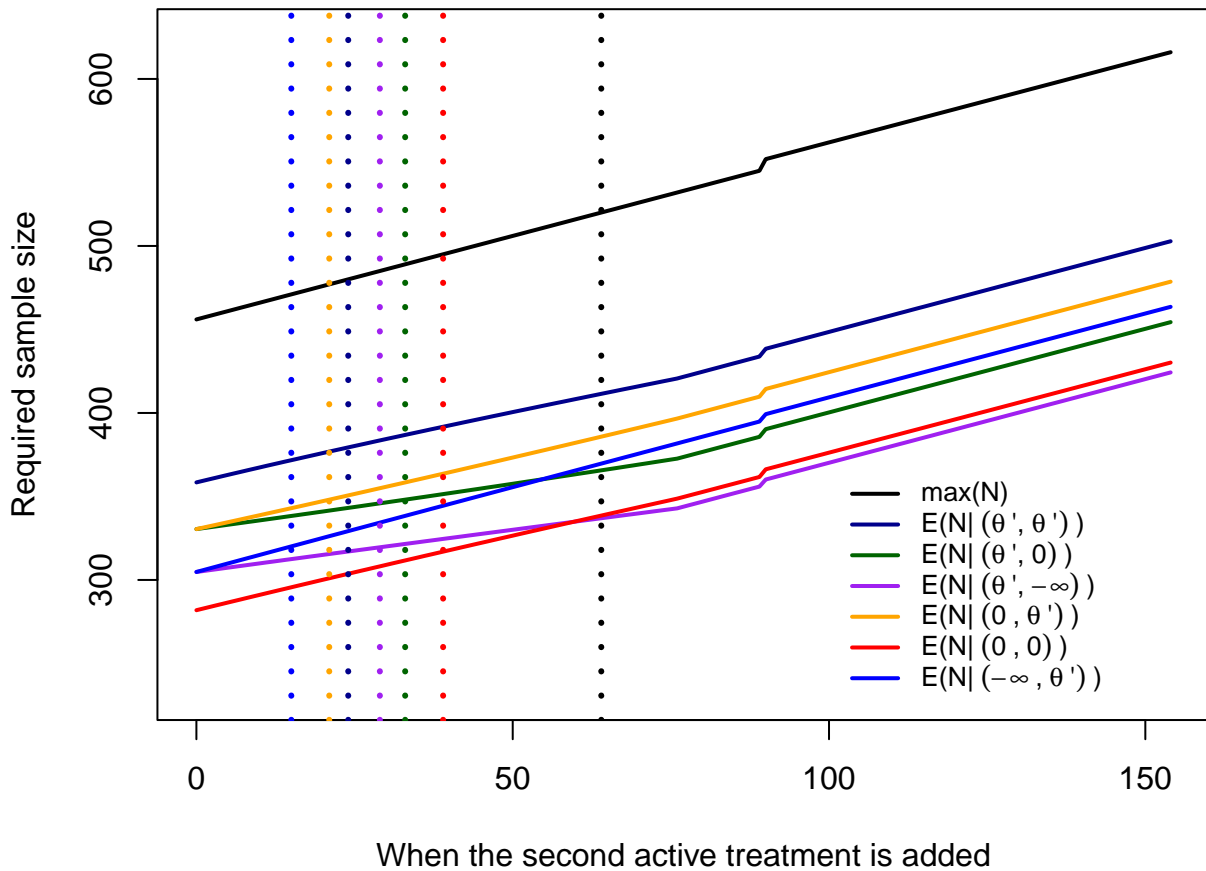

Supplement: Supplementary file 1 — Supporting Information [file BIMJ-67-e70025-s001.zip › Code supplement/Fig1b.pdf]

## Setting 2

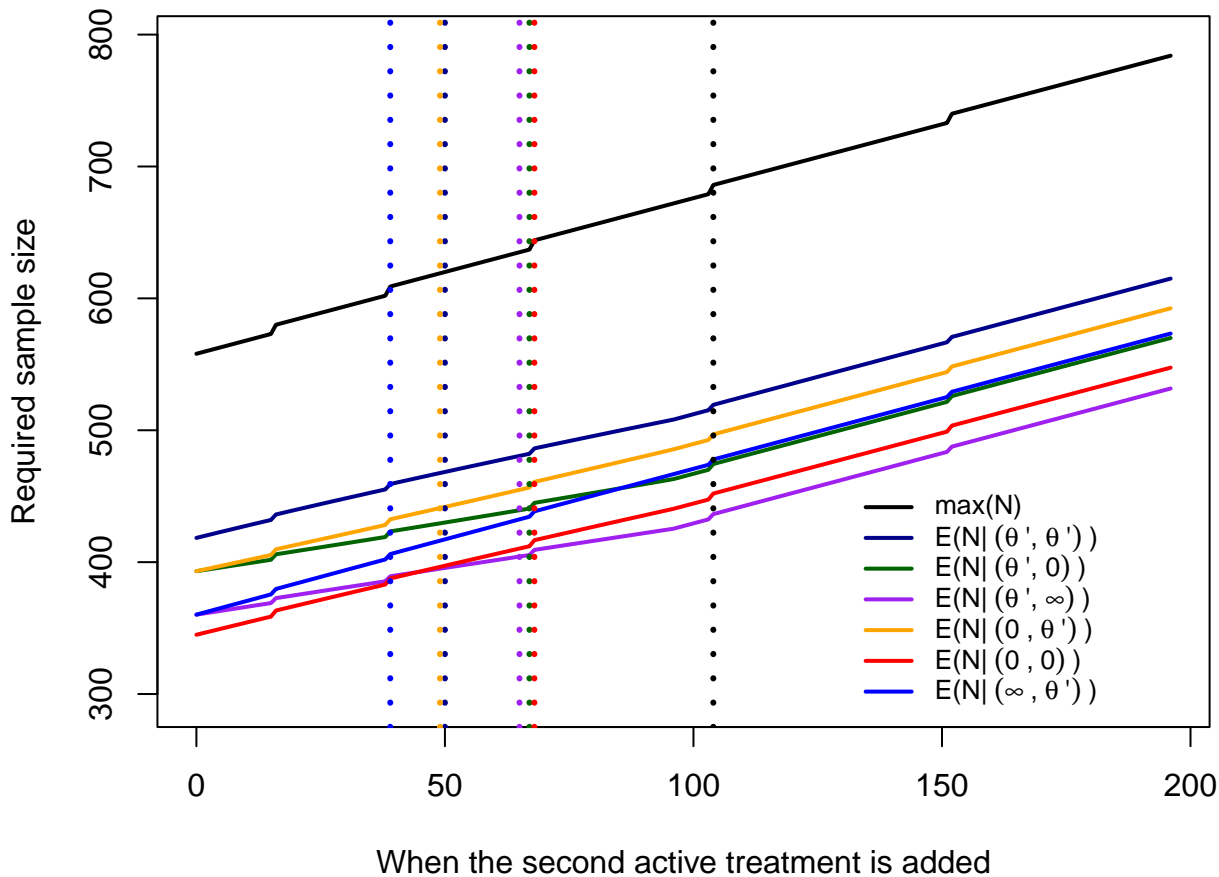

Supplement: Supplementary file 1 — Supporting Information [file BIMJ-67-e70025-s001.zip › Code supplement/Fig2b.pdf]

## Setting 1

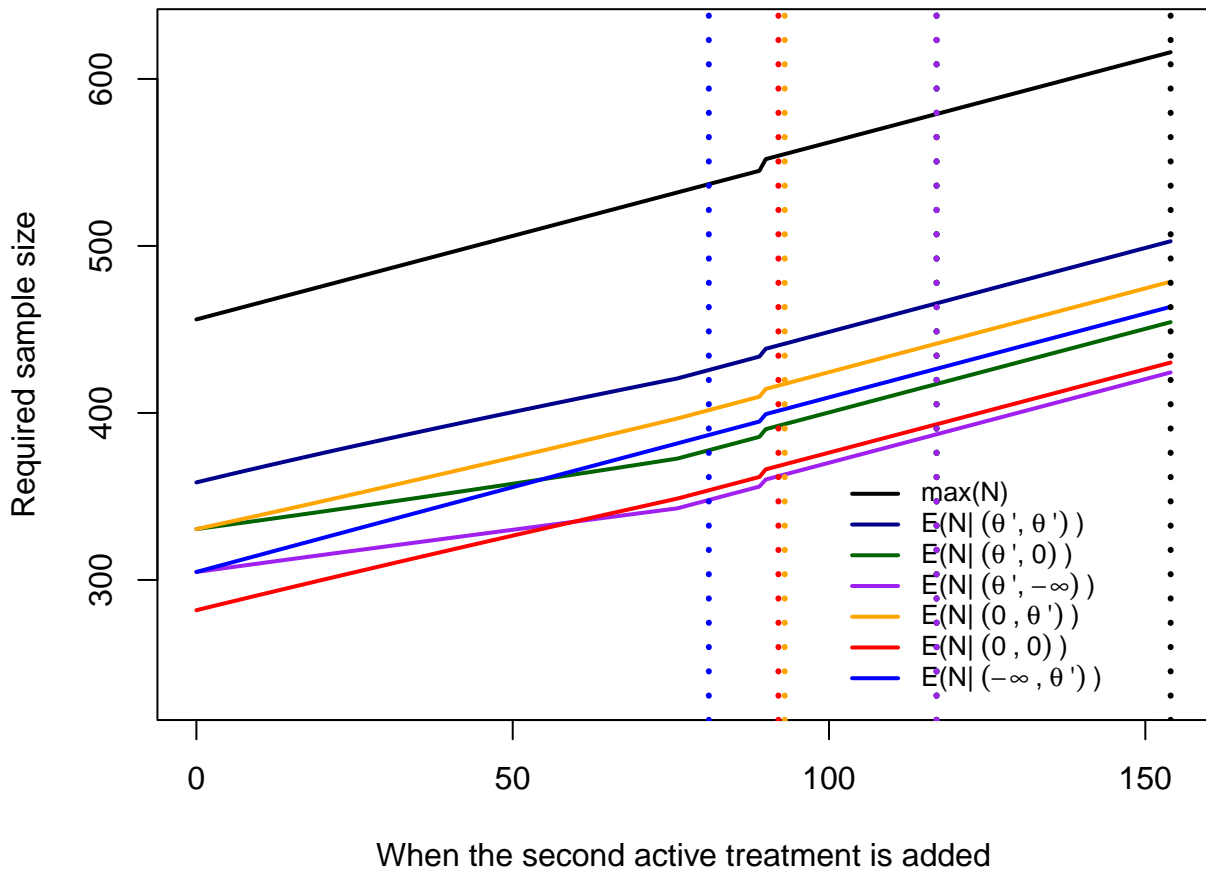

Supplement: Supplementary file 1 — Supporting Information [file BIMJ-67-e70025-s001.zip › Code supplement/Fig1a.pdf]

## Setting 1

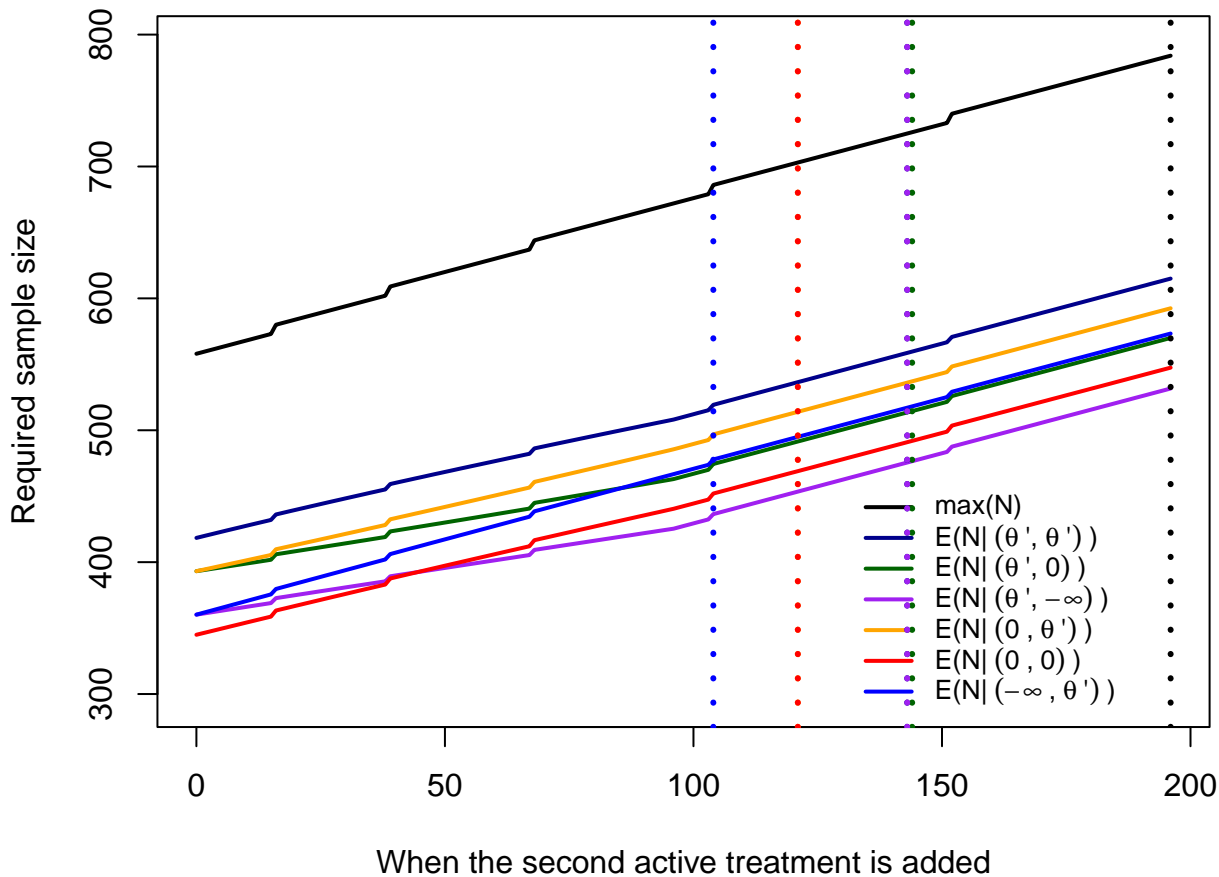

Supplement: Supplementary file 1 — Supporting Information [file BIMJ-67-e70025-s001.zip › Code supplement/Fig2a.pdf]

## Pairwise Power Setting 2

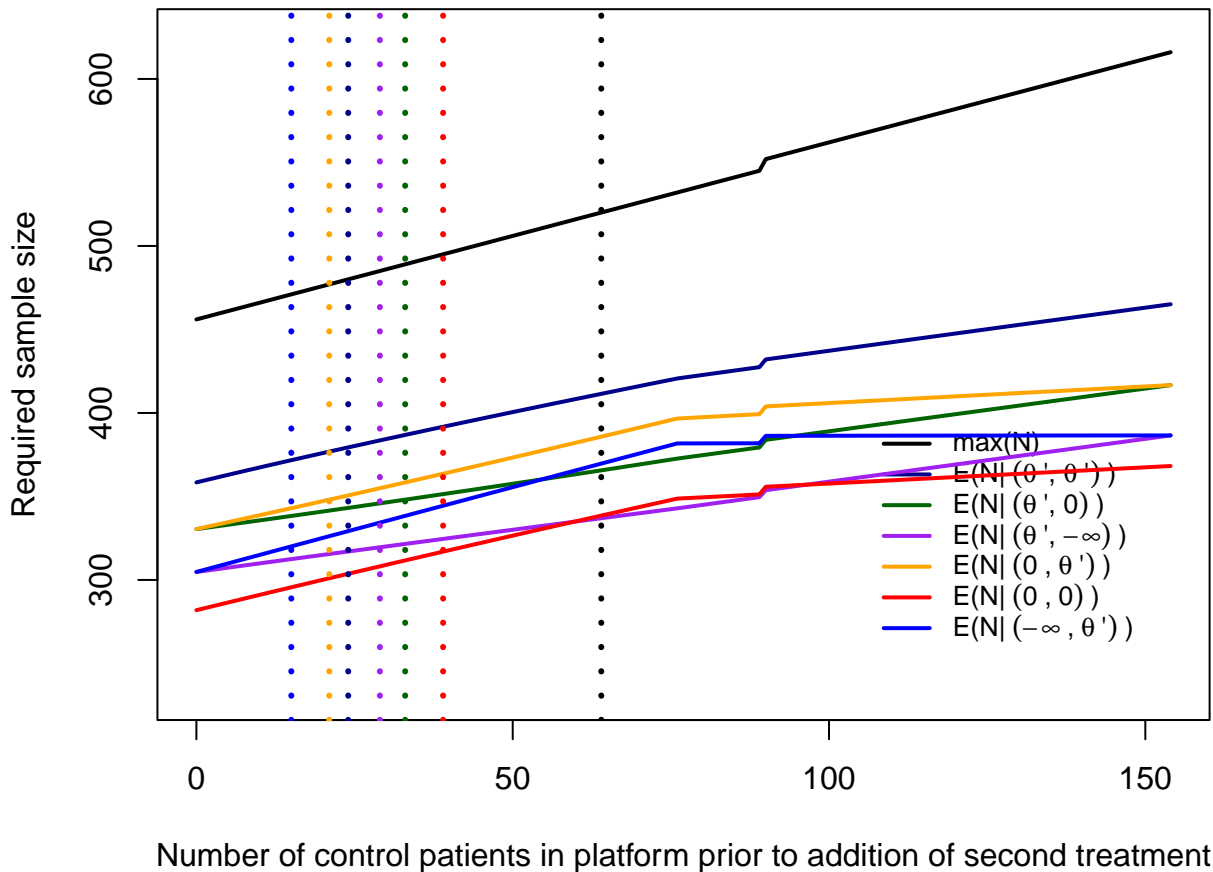

Supplement: Supplementary file 1 — Supporting Information [file BIMJ-67-e70025-s001.zip › Code supplement/Fig3a.pdf]

## Conjunctive Power Setting 2

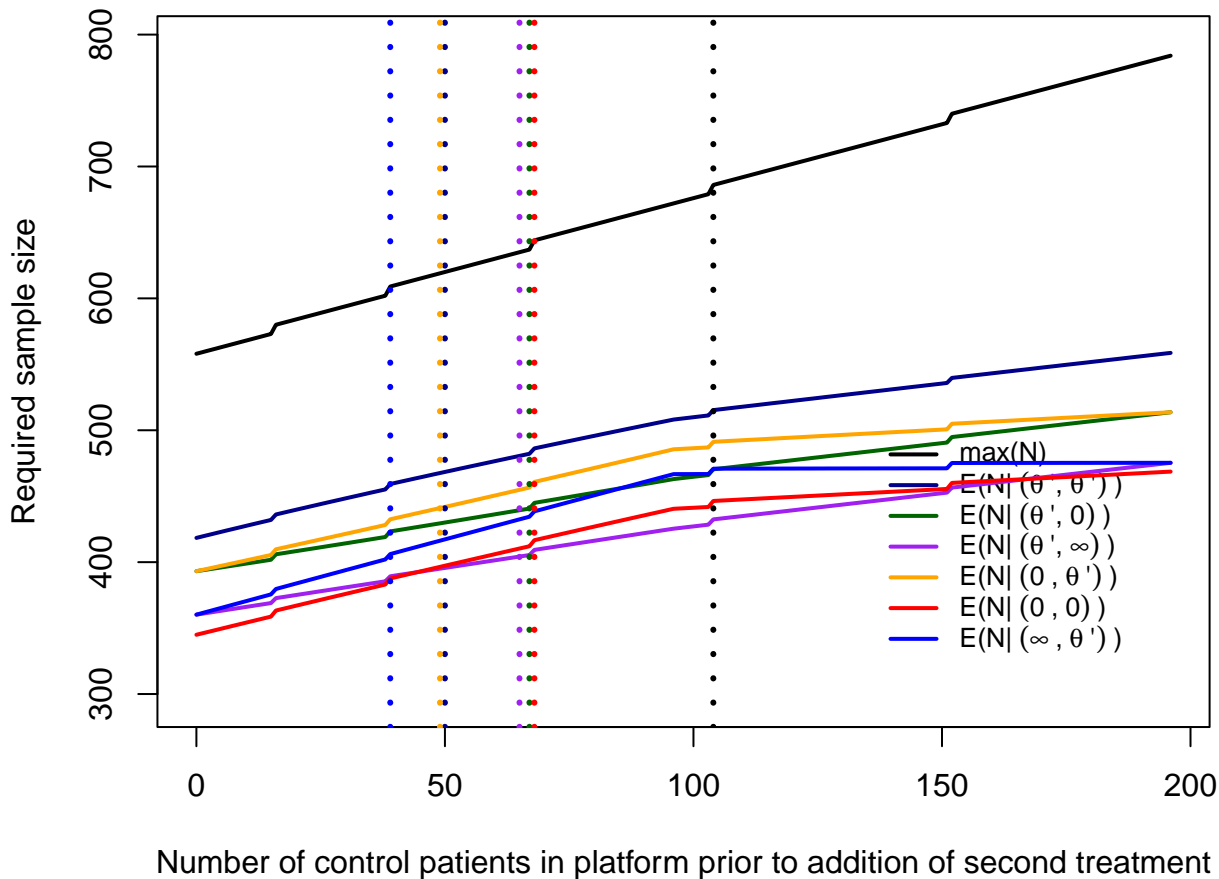

Supplement: Supplementary file 1 — Supporting Information [file BIMJ-67-e70025-s001.zip › Code supplement/Fig3b.pdf]

### 3 arm 2 stage trial example

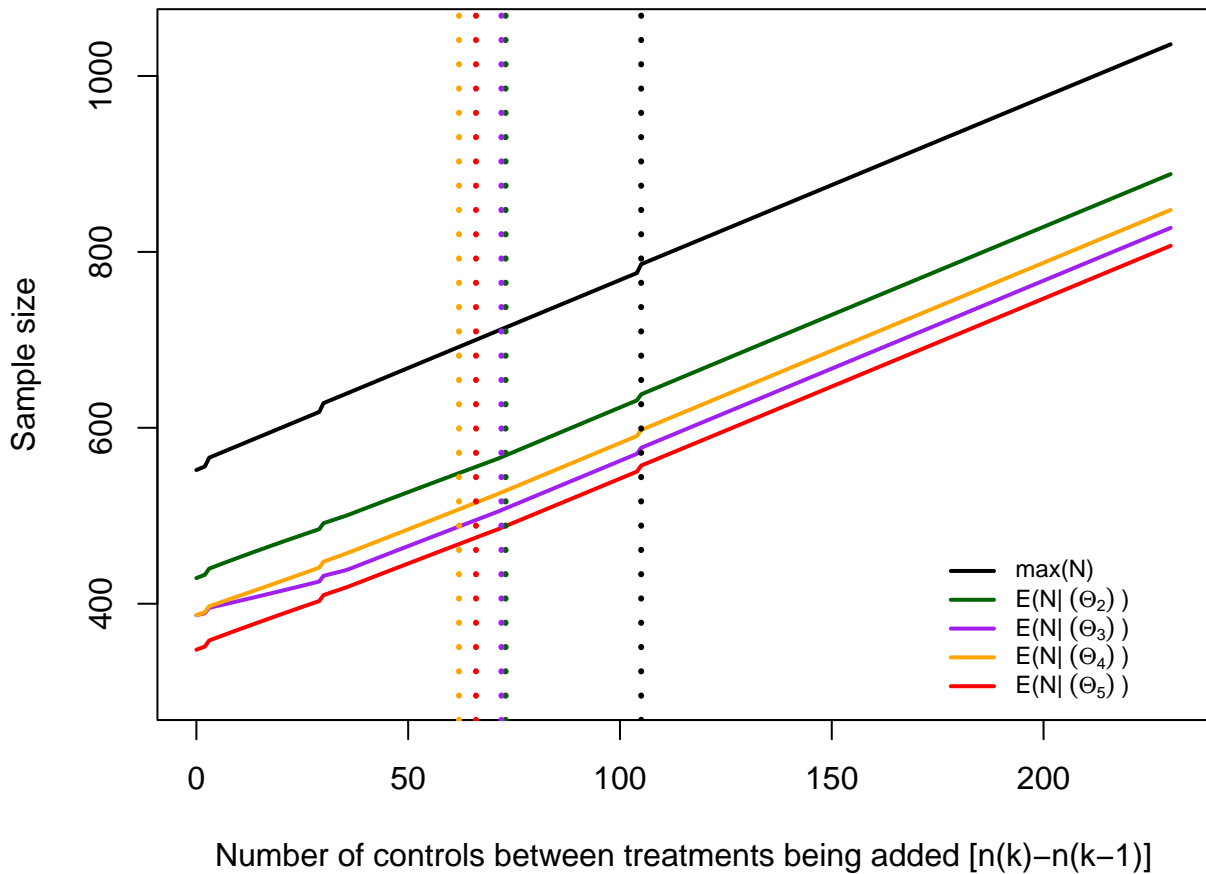

Supplement: Supplementary file 1 — Supporting Information [file BIMJ-67-e70025-s001.zip › Code supplement/SIFig1a.pdf]

### 3 arm 3 stage trial example

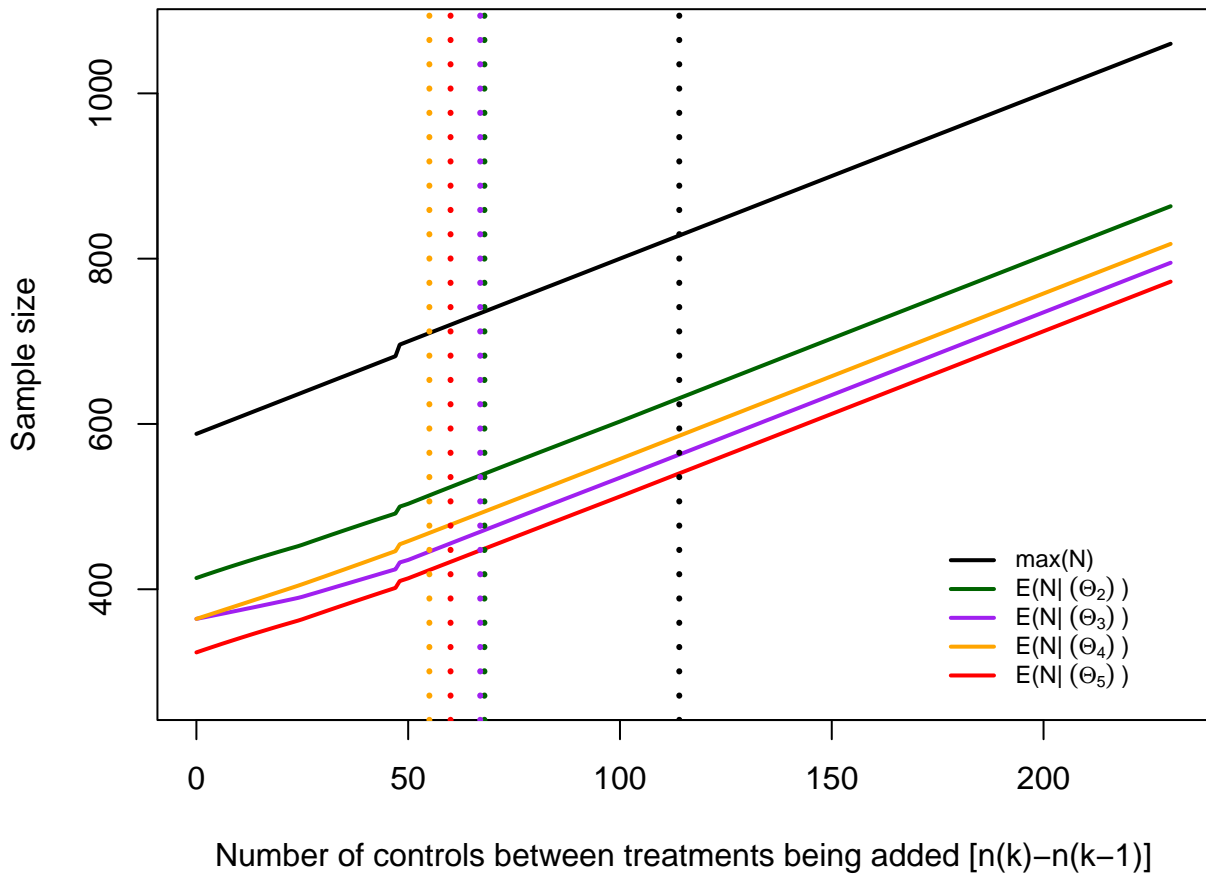

Supplement: Supplementary file 1 — Supporting Information [file BIMJ-67-e70025-s001.zip › Code supplement/SIFig1b.pdf]

## 4 arm 2 stage trial example

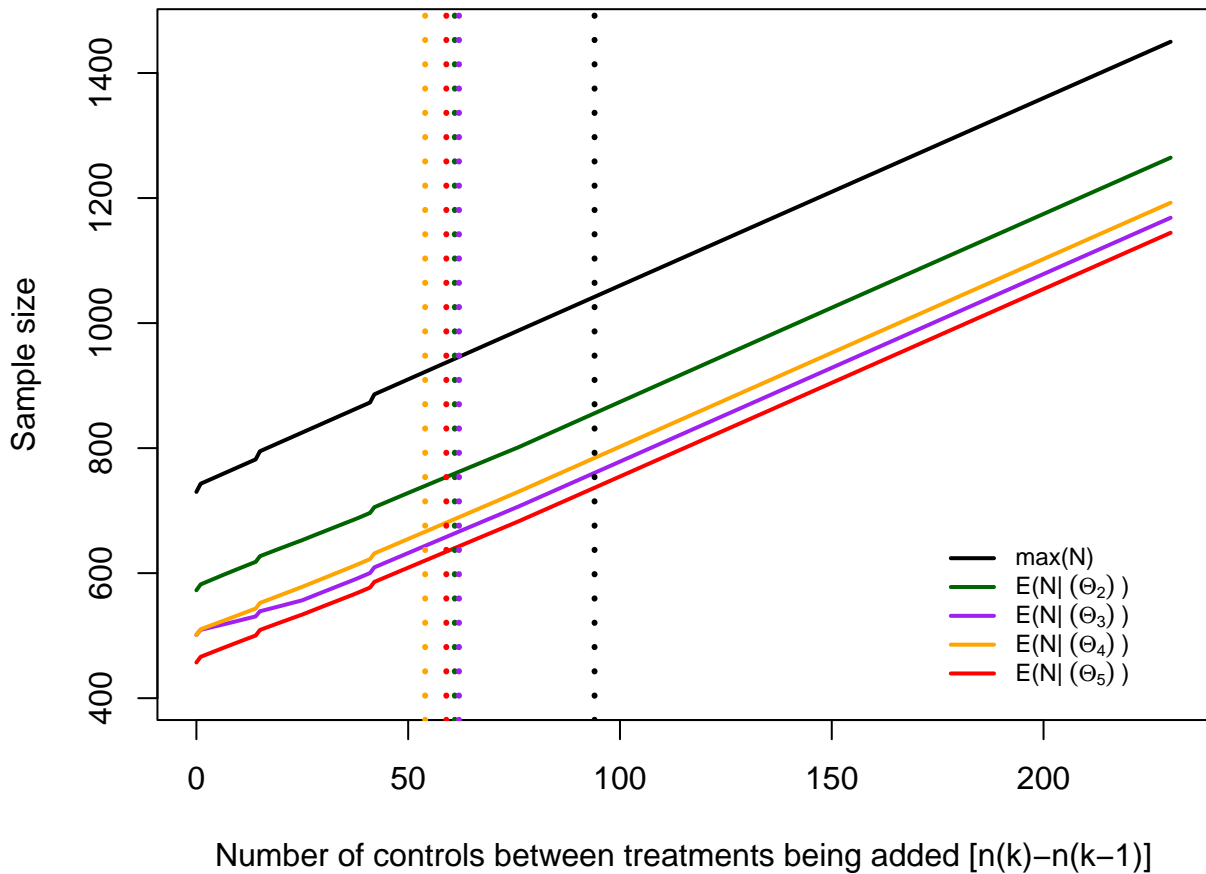

Supplement: Supplementary file 1 — Supporting Information [file BIMJ-67-e70025-s001.zip › Code supplement/SIFig1c.pdf]

## 4 arm 3 stage trial example

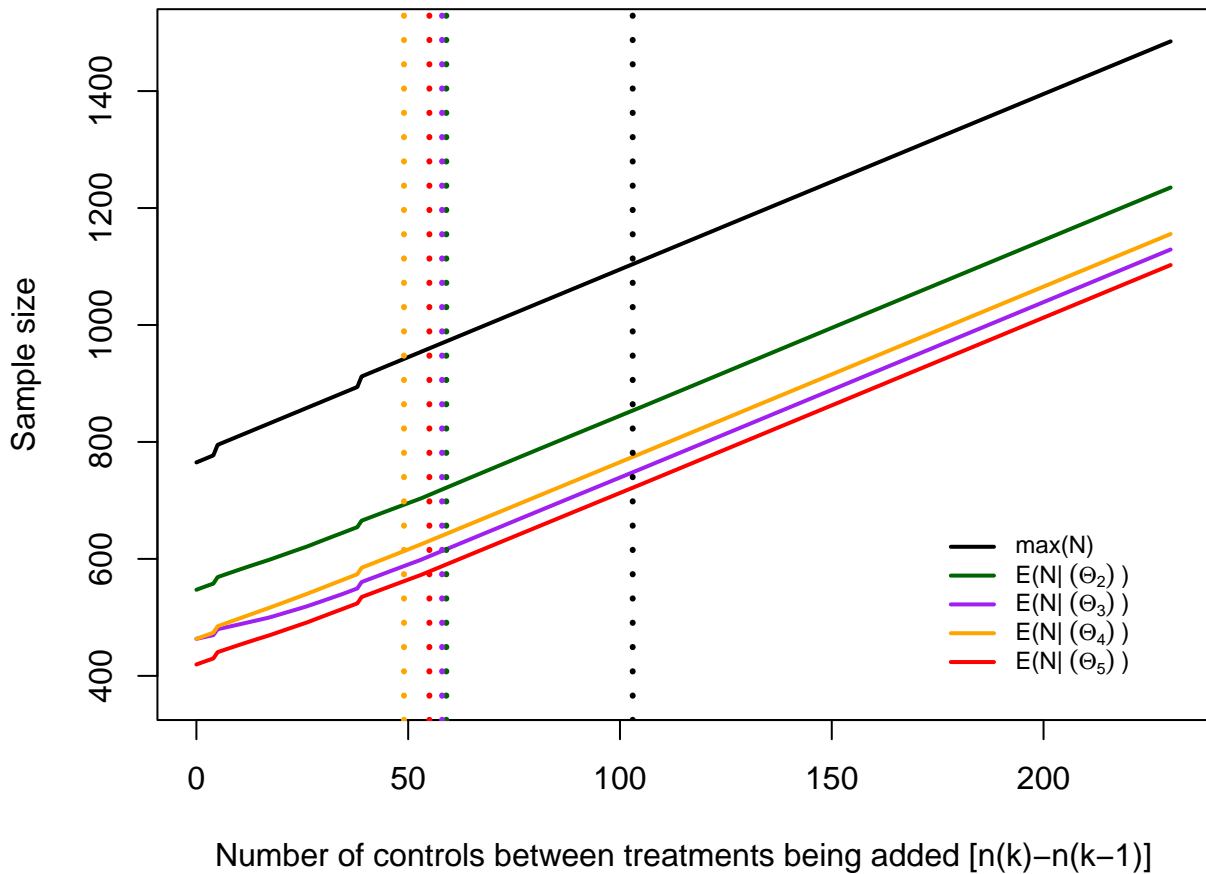

Supplement: Supplementary file 1 — Supporting Information [file BIMJ-67-e70025-s001.zip › Code supplement/SIFig1d.pdf]

### 3 arm 2 stage trial example

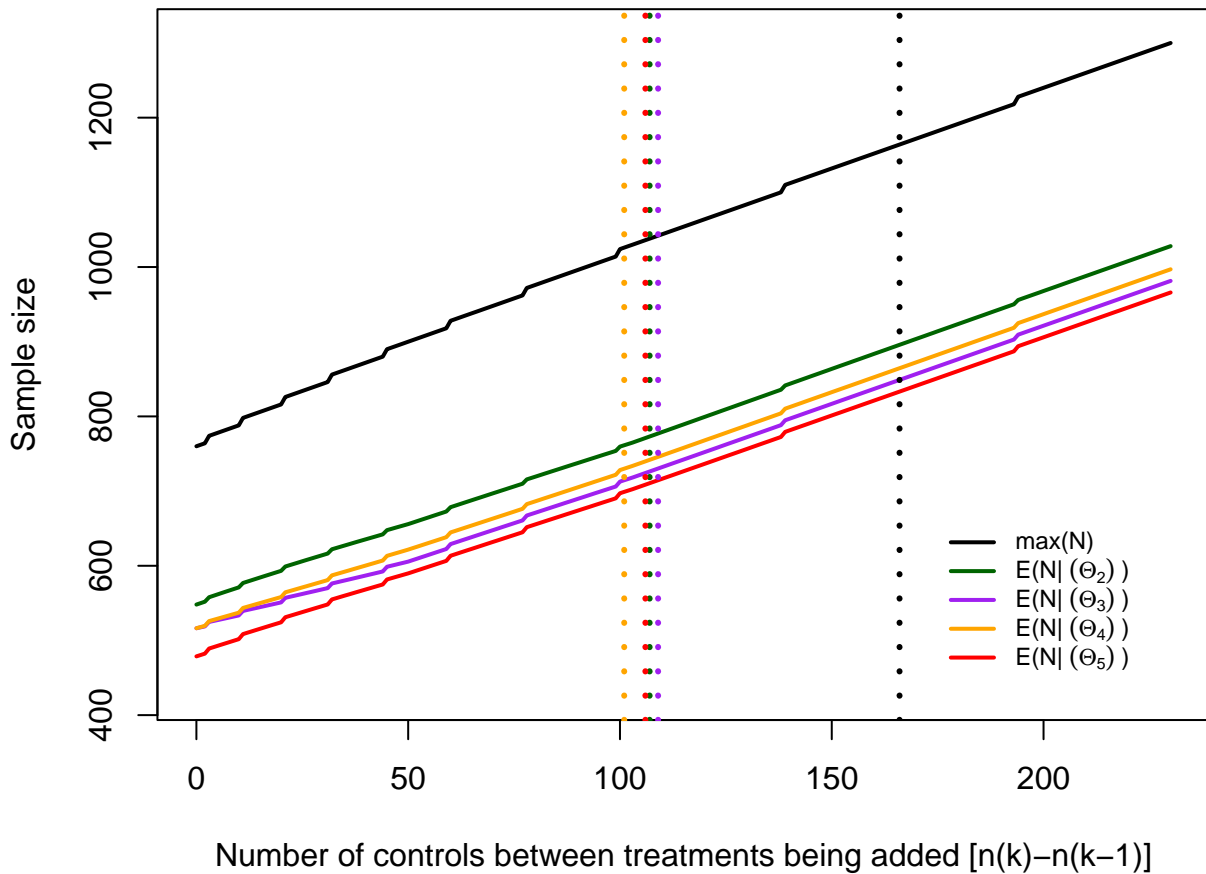

Supplement: Supplementary file 1 — Supporting Information [file BIMJ-67-e70025-s001.zip › Code supplement/SIFig2a.pdf]

### 3 arm 3 stage trial example

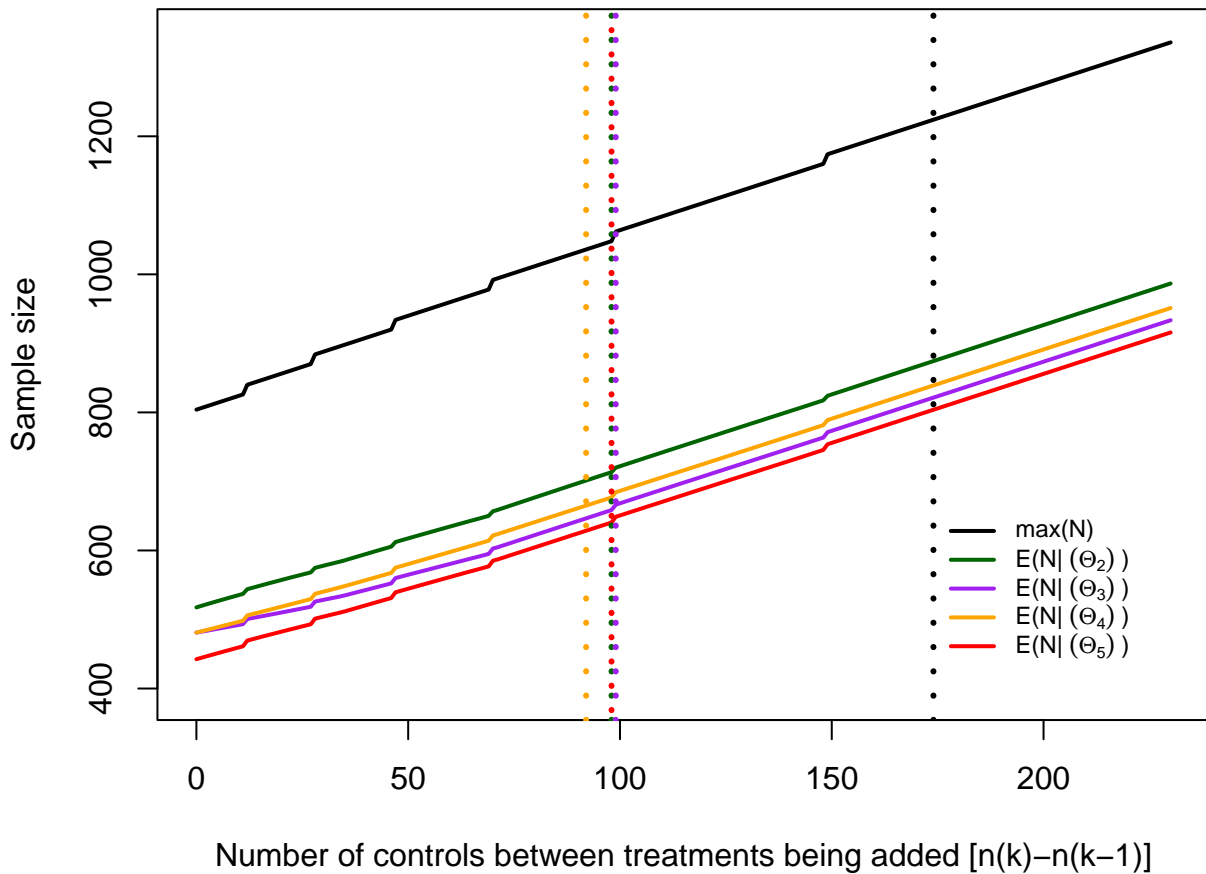

Supplement: Supplementary file 1 — Supporting Information [file BIMJ-67-e70025-s001.zip › Code supplement/SIFig2b.pdf]

## 4 arm 2 stage trial example

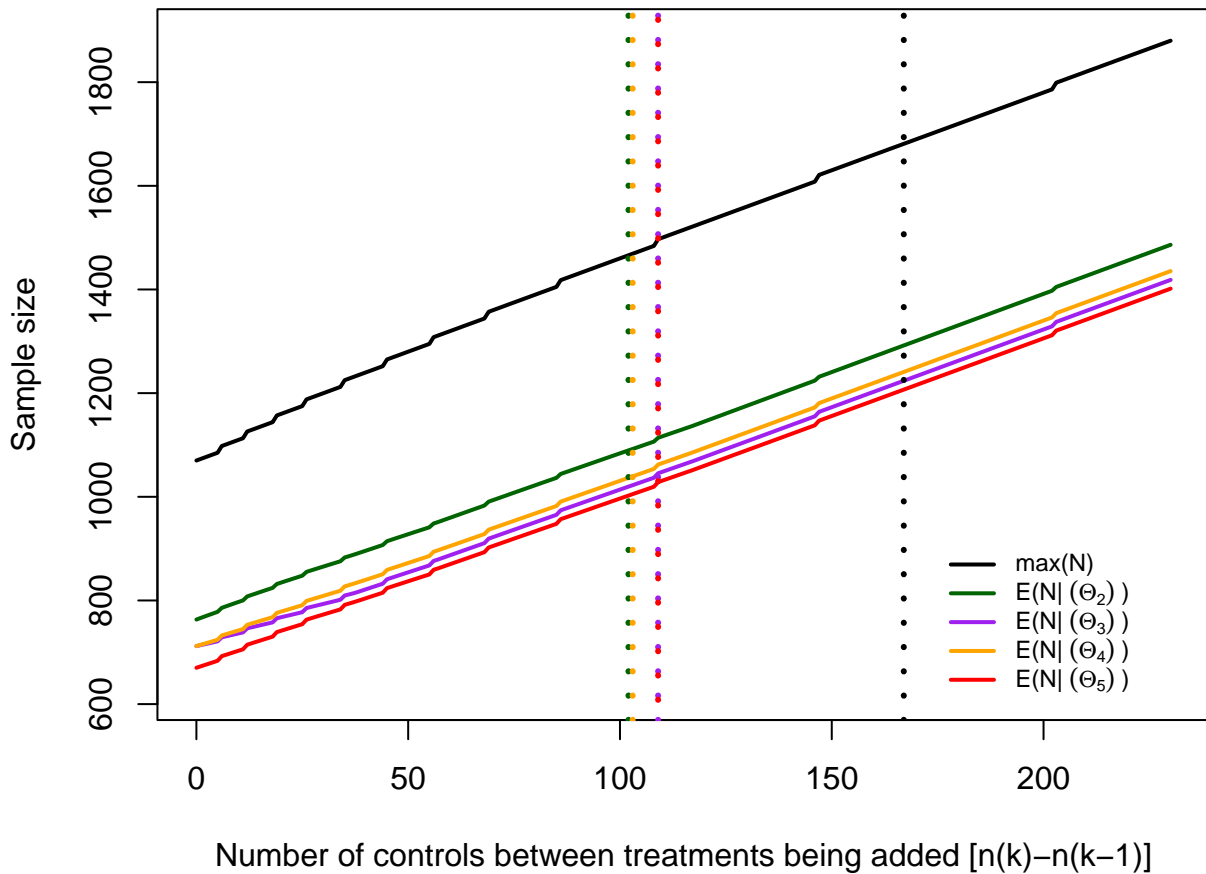

Supplement: Supplementary file 1 — Supporting Information [file BIMJ-67-e70025-s001.zip › Code supplement/SIFig2c.pdf]

## 4 arm 3 stage trial example

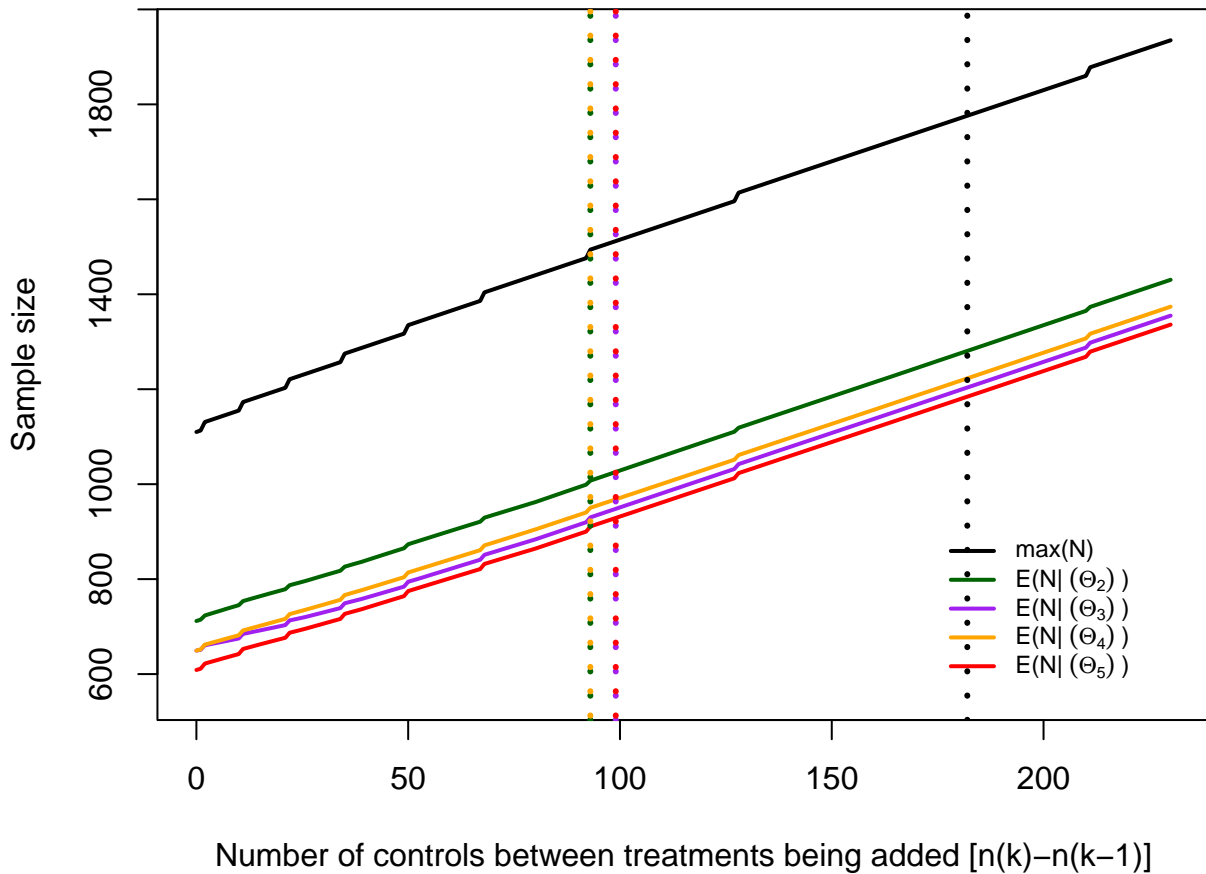

Supplement: Supplementary file 1 — Supporting Information [file BIMJ-67-e70025-s001.zip › Code supplement/SIFig2d.pdf]
