# Supplementary material for: A Preplanned Multi‐Stage Platform Trial for Discovering Multiple Superior Treatments With Control of FWER and Power
Source: Biom J. 2024 Dec 22;67(1):e70025. doi: 10.1002/bimj.70025 (PMC11664203; doi:10.1002/bimj.70025)
Supplement: Supplementary file 2 — Supporting Information [file BIMJ-67-e70025-s002.pdf]

Biometrical Journal **52** (2010) 61, zzz–zzz / DOI: 10.1002/bimj.200100000

Supporting information: A preplanned multi-stage platform trial for discovering multiple superior treatments with control of FWER and power

Peter Greenstreet[*] [1,2], **Thomas Jaki** [3,4], **Alun Bedding** [5], and **Pavel Mozgunov** [3]

[1] Department of Mathematics and Statistics, Lancaster University, Lancaster, UK
[2] Exeter Clinical Trials Unit, University of Exeter, Exeter, UK
[3] MRC Biostatistics Unit, University of Cambridge, Cambridge, UK
[4] University of Regensburg, Regensburg, Germany
[5] Roche Products Ltd., Welwyn Garden City, UK

Received zzz, revised zzz, accepted zzz

1 Tables of notation introduced in the paper

In Table S1 the notation defined in Section 2.1 is given; in Table S2 the notation defined in Section 2.2 is given; in Table S3 the notation defined in Section 2.3 is given and in Table S4 the notation defined in Section 2.4 is given.

2 Proof of FWER

As in Magirr et al. (2012) we define for any vector of constants $\Theta = (\theta_1, \ldots, \theta_K)$ and $k = 1, \ldots, K$, $j_k = 1, \ldots, J_k$, then define the events,

$$A_{k,j}(\theta_k) =[Z_{k,j} < l_{k,j} + (\mu_k - \mu_0 - \theta_k)I_{k,j}^{1/2}],$$
$$B_{k,j}(\theta_k) =[l_{k,j} + (\mu_k - \mu_0 - \theta_k)I_{k,j}^{1/2} < Z_{k,j} < u_{k,j} + (\mu_k - \mu_0 - \theta_k)I_{k,j}^{1/2}].$$

The event that $H_{01}, ..., H_{0K}$ all fail to be rejected is equal to

$$1 - P(\bar{R}_K(\Theta)) = 1 - P\Big(\bigcap_{k\in\{m_1,\ldots,m_K\}} \left(\bigcup_{j_k=1}^{J_k} \left[\left(\bigcap_{j=1}^{j_k-1} B_{k,j}(\Theta) \right) \cap A_{k,j_k}(\Theta) \right] \right)\Big)$$

where if $\mu_k - \mu_0 = \theta_k$ for $k = 1, \ldots, K$, the event that $H_{01}, \ldots, H_{0K}$ all fail to be rejected is equal to $\bar{R}_K(\Theta)$. The convention that $\bigcap_{i=1}^{0} = \Omega$ where Ω is the whole sample space is used and $m_1 \in \{1, \ldots, K\}$ and $m_k \in \{1, \ldots, K\}\backslash\{m_1, \ldots, m_{k-1}\}$. Therefore $\{m_1, \ldots, m_K\} = \{1, \ldots, K\}$. This notation reflects the fact that the order in which treatments are added affects the FWER as seen in Greenstreet et al. (2024).

Theorem 2.1 *For any Θ, under the conditions above, $P($reject at least one true $H_{0k}|\Theta) \leq P($reject at least one true $H_{0k}|H_G)$.*

*Corresponding author: e-mail: peterjgreenstreet@gmail.com

© 2010 WILEY-VCH Verlag GmbH & Co. KGaA, Weinheim

Table S1 Notation defined in Section 2.1

Notation	Definition
K	Number of experimental arms.
$K^\star$	Number of experimental arms beginning the trial.
J_k	Maximum number of stages for treatment k.
σ^2	Variance of primary outcome measure.
$n(k)$	Number of patients recruited to the control treatment before treatment k starts.
$\mathbf{n}(\mathbf{K})$	$\mathbf{n}(\mathbf{K}) = (n(1), \dots, n(K))$.
$n_{k,j}$	Number of patients recruited to treatment k by the end of its jth stage.
n_k	$n_k = n_{k,1}$.
$n_{0,k,j}$	Number of patients recruited to the control at the end of treatment k's j^{th} stage.
N	Total sample size of a trial.
$r(k)$	$r(k) = n(k)/n_{1,1}$.
$r_{k,j}$	$r_{k,j} = n_{k,j}/n_{1,1}$.
$r_{0,k,j}$	$r_{0,k,j} = n_{0,k,j}/n_{1,1}$.
μ_k	Mean responses on treatment k.
H_{0k}	Null hypotheses of interest, $H_{0k} : \mu_k \leq \mu_0$.
H_G	Global null hypothesis, $\mu_0 = \mu_1 = \mu_2 = \ldots = \mu_K$.
$X_{k,i}$	Response from patient i on treatment k.
$Z_{k,j}$	Test statistic for treatment k's j^{th} stage.
$u_{k,j}$	Upper boundary for treatment k's j^{th} stage test statistic.
$l_{k,j}$	Lower boundary for treatment k's j^{th} stage test statistic.
U_k	$U_k = (u_{k,1}, \dots, u_{k,J_k})$.
L_k	$L_k = (l_{k,1}, \dots, l_{k,J_k})$.

Table S2 Notation defined in Section 2.2

Notation	Definition
α	Desired level of control for the FWER.
j_k	The stage treatment k stops.
$U_{k,j_k}(0)$	$U_{k,j_k}(0) = (u_{k,1}, \dots, l_{k,j_k})$.
$L_{k,j_k}(0)$	$L_{k,j_k}(0) = (l_{k,1}, \dots, -\infty)$.
$\mathbf{j_k}$	$\mathbf{j_k} = (j_1, \dots, j_K)$.
$\Phi(L, U, \Sigma)$	Multivariate standard normal distribution function with mean zero and covariance matrix Σ between the lower boundaries L and upper boundaries U.
$\mathbf{U_{j_k}(0)}$	$\mathbf{U_{j_k}(0)} = (U_{1,j_1}(0), \dots, U_{K,j_K}(0))$.
$\mathbf{L_{j_k}(0)}$	$\mathbf{L_{j_k}(0)} = (L_{1,j_1}(0), \dots, L_{K,j_K}(0))$.
$\Sigma_{\mathbf{j_k}}$	Correlation matrix used for calculating FWER.
$\rho_{(k,j),(k^\star,j^\star)}$	Each elements in $\Sigma_{\mathbf{j_k}}$ for given treatments $k, k^\star$ and stages $j, j^\star$.
a	Single scalar parameter a used in the boundary functions.
$g(a)$	Function for the shape of the upper boundaries.
$f(a)$	Function for the shape of the lower boundaries.

Proof. If $\mu_k - \mu_0 = \theta_k$ for $k = 1, \dots, K$, the event that $H_{01}, \dots, H_{0K}$ all fail to be rejected is equivalent to

$$\bar{R}_K(\Theta) = \bigcap_{k \in \{m_1, \dots, m_K\}} \left(\bigcup_{j_k=1}^{J_k} \left[\left(\bigcap_{i=1}^{j_k-1} B_{k,j}(\theta_k) \right) \cap A_{k,j_k}(\theta_k) \right] \right).$$

© 2010 WILEY-VCH Verlag GmbH & Co. KGaA, Weinheim

Table S3 Notation defined in Section 2.3

Notation	Definition
$1-\beta$	Chosen value for power to be greater than or equal to.
P_D	Disjunctive power.
$P_{pw,k}$	Pairwise power for treatment k.
θ_k	$\theta_k = \mu_k - \mu_0$.
$I_{k,j}$	$I_{k,j} = \sigma^2(n_{k,j}^{-1} + (n_{0,k,j} - n(k))^{-1})$.
θ'	Clinically relevant effect.
$L_{k,j}^{+}(\theta_k)$	$L_{k,j}^{+}(\theta_k) = (l_{k,1} - \frac{\theta_k}{\sqrt{I_{k,1}}}, \ldots, l_{k,j-1} - \frac{\theta_k}{\sqrt{I_{k,j-1}}}, u_{k,j} - \frac{\theta_k}{\sqrt{I_{k,j}}})$.
$U_{k,j}^{+}(\theta_k)$	$U_{k,j}^{+}(\theta_k) = (u_{k,1} - \frac{\theta_k}{\sqrt{I_{k,1}}}, \ldots, u_{k,j-1} - \frac{\theta_k}{\sqrt{I_{k,j-1}}}, \infty)$.
$\ddot{\Sigma}_{k,j_k}$	Correlation matrix used for calculating $P_{pw,k}$.
n	If $n_k = n_{k^\star}$ for all $k, k^\star \in 1, \ldots, K$ then $n = n_k$.
$B_{k,j}(\theta_k)$	$B_{k,j}(\theta_k) = [l_{k,j} + (\mu_k - \mu_0 - \theta_k)I_{k,j}^{1/2} < Z_{k,j} < u_{k,j} + (\mu_k - \mu_0 - \theta_k)I_{k,j}^{1/2}]$.
$C_{k,j}(\theta_k)$	$C_{k,j}(\theta_k) = [Z_{k,j} > u_{k,j} + (\mu_k - \mu_0 - \theta_k)I_{k,j}^{1/2}]$.
Θ	$\Theta = (\theta_1, \theta_2, \ldots, \theta_K)$.
$\bar{W}_K(\Theta)$	If $\mu_k - \mu_0 = \theta_k$ for $k = 1, \ldots, K$, $\bar{W}_K(\Theta)$ is the event that $H_{01}, \ldots, H_{0K}$ are all rejected.
m_k	$m_k \in \{1, \ldots, K\} \backslash \{m_1, \ldots, m_{k-1}\}$.
Ω	Whole sample space.
Θ'	$\Theta' = (\theta', \ldots, \theta')$.
P_C	Conjunctive power.
$\mathbf{U}_{\mathbf{j_k}}^{+}(\Theta')$	$\mathbf{U}_{\mathbf{j_k}}^{+}(\Theta') = (U_{1,j_1}^{+}(\theta'), \ldots, U_{K,j_K}^{+}(\theta'))$.
$\mathbf{L}_{\mathbf{j_k}}^{+}(\Theta')$	$\mathbf{L}_{\mathbf{j_k}}^{+}(\Theta') = (L_{1,j_1}^{+}(\theta'), \ldots, L_{K,j_K}^{+}(\theta'))$.

Table S4 Notation defined in Section 2.4

Notation	Definition
q_k	$q_k = 0$ indicates that treatment k falls below the lower stopping boundary at point j_k, and $q_k = 1$ indicates that treatment k exceeds the upper stopping boundary at point j_k.
$\mathbf{q_k}$	$\mathbf{q_k} = (q_1, \ldots, q_K)$
$Q_{\mathbf{j_k},\mathbf{q_k}}$	Probability for each outcome of the trial.
$\tilde{L}_{k,j,q_k}(\theta_k)$	$\tilde{L}_{k,j,q_k}(\theta_k) = (l_{k,1} - \frac{\theta_k}{\sqrt{I_{k,1}}}, \ldots, l_{k,j-1} - \frac{\theta_k}{\sqrt{I_{k,j-1}}}, [\mathbb{1}(q_k = 0)(-\infty) + u_{k,j}] - \frac{\theta_k}{\sqrt{I_{k,j}}})$.
$\tilde{U}_{k,j,q_k}(\theta_k)$	$\tilde{U}_{k,j,q_k}(\theta_k) = (u_{k,1} - \frac{\theta_k}{\sqrt{I_{k,1}}}, \ldots, u_{k,j-1} - \frac{\theta_k}{\sqrt{I_{k,j-1}}}, [\mathbb{1}(q_k = 1)(\infty) + l_{k,j}] - \frac{\theta_k}{\sqrt{I_{k,j}}})$.
$\tilde{\mathbf{L}}_{\mathbf{j_k},\mathbf{q_k}}(\Theta)$	$\tilde{\mathbf{L}}_{\mathbf{j_k},\mathbf{q_k}}(\Theta) = (\tilde{L}_{1,j_1,q_1}(\theta_1), \ldots, \tilde{L}_{K,j_K,q_K}(\theta_K))$.
$\tilde{\mathbf{U}}(\Theta)_{\mathbf{j_k},\mathbf{q_k}}$	$\tilde{\mathbf{U}}(\Theta)_{\mathbf{j_k},\mathbf{q_k}} = (\tilde{U}_{1,j_1,q_1}(\theta_1), \ldots, \tilde{U}_{K,j_K,q_K}(\theta_K))$.
$N_{\mathbf{j_k},\mathbf{q_k}}$	Trial sample size for a given $Q_{\mathbf{j_k},\mathbf{q_k}}$.
$E(N\|\Theta)$	Expected sample size of the trial for a given Θ.

Then for any $\epsilon_k > 0$,

$$\bigcup_{j_k=1}^{J_k}\left[\left(\bigcap_{j=1}^{j_k-1} B_{k,j}(\theta_k+\epsilon_k)\right)\cap A_{k,j_k}(\theta_k+\epsilon_k)\right] \subseteq \bigcup_{j_k=1}^{J_k}\left[\left(\bigcap_{j=1}^{j_k-1} B_{k,j}(\theta_k)\right)\cap A_{k,j_k}(\theta_k)\right].$$

Take any

$$w = (Z_{k,1}, \ldots, Z_{k,J_k}) \in \bigcup_{j_k=1}^{J_k}\left[\left(\bigcap_{j=1}^{j_k-1} B_{k,j}(\theta_k+\epsilon_k)\right)\cap A_{k,j_k}(\theta_k+\epsilon_k)\right].$$

© 2010 WILEY-VCH Verlag GmbH & Co. KGaA, Weinheim

For some $j_k \in \{1,\dots,J_k\}$, for which $Z_{k,j_k} \in A_{k,j_k}(\theta_k+\epsilon_k)$ and $Z_{k,j} \in B_{k,j}(\theta_k+\epsilon_k)$ for $j = 1,\dots,j_k-1$. $Z_{k,j_k} \in A_{k,j_k}(\theta_k+\epsilon_k)$ implies that $Z_{k,j_k} \in A_{k,j_k}(\theta_k)$. Furthermore $Z_{k,j_k} \in B_{k,j_k}(\theta_k+\epsilon_k)$ implies that $Z_{k,j_k} \in B_{k,j_k}(\theta_k) \cup A_{k,q}(\theta_k)$ for some $j = 1,\dots,j_k-1$. Therefore,

$$w \in \bigcup_{j_k=1}^{J_k} \left[\left(\bigcap_{j=1}^{j_k-1} B_{k,j}(\theta_k) \right) \cap A_{k,j_k}(\theta_k) \right].$$

Next suppose for any $m_1,\dots,m_K$ where $m_1 \in \{1,\dots,K\}$ and $m_k \in \{1,\dots,K\}\backslash\{m_1,\dots,m_{k-1}\}$ with $\theta_{m_1},\dots,\theta_{m_l} \le 0$ and $\theta_{m_{l+1}},\dots,\theta_{m_K} > 0$. Let $\Theta_l = (\theta_{m_1},\dots,\theta_{m_l})$. Then

$$\begin{aligned}
&P(\text{reject at least one true } H_{0k}|\Theta) \\
&\quad = 1 - P(\bar{R}_l(\Theta_l)) \\
&\quad \le 1 - P(\bar{R}_l(0)) \\
&\quad \le 1 - P(\bar{R}_K(0)) \\
&\quad = P(\text{reject at least one true } H_{0k}|H_G).
\end{aligned}$$

□

The following proof was nearly identical to the one presented in Greenstreet et al. (2024) and builds on the work of Magirr et al. (2012). The only change from Greenstreet et al. (2024) is now is $P(\text{reject at least one true } H_{0k}|\Theta) = 1 - P(\bar{R}_l(\Theta_l))$ instead of being $P(\text{reject at least one true } H_{0k}|\Theta) \le 1 - P(\bar{R}_l(\Theta_l))$.

3 The correlation matrix $\Sigma_{\mathbf{j_k}}$

As introduced in Section 2.2, $\Sigma_{\mathbf{j_k}}$ is the correlation matrix used when calculating the FWER, disjunctive power and conjunctive power. The correlation structure is

$$\Sigma_{\mathbf{j_k}} = \begin{pmatrix}
\rho_{(1,1),(1,1)} & \rho_{(1,1),(1,2)} & \cdots & \rho_{(1,1),(1,j_1)} & \rho_{(1,1),(2,1)} & \cdots & \rho_{(1,1),(K,j_k)} \\
\rho_{(1,2),(1,1)} & \rho_{(1,2),(1,2)} & \cdots & \rho_{(1,2),(1,j_1)} & \rho_{(1,2),(2,1)} & \cdots & \rho_{(1,2),(K,j_k)} \\
\vdots & \vdots & \ddots & \vdots & \vdots & \ddots & \vdots \\
\rho_{(1,j_1),(1,1)} & \rho_{(1,j_1),(1,2)} & \cdots & \rho_{(1,j_1),(1,j_1)} & \rho_{(1,j_1),(2,1)} & \cdots & \rho_{(1,j_1),(K,j_k)} \\
\rho_{(2,1),(1,1)} & \rho_{(2,1),(1,2)} & \cdots & \rho_{(2,1),(1,j_1)} & \rho_{(2,1),(2,1)} & \cdots & \rho_{(2,1),(K,j_k)} \\
\vdots & \vdots & \ddots & \vdots & \vdots & \ddots & \vdots \\
\rho_{(K,j_k),(1,1)} & \rho_{(K,j_k),(1,2)} & \cdots & \rho_{(K,j_k),(1,j_1)} & \rho_{(K,j_k),(2,1)} & \cdots & \rho_{(K,j_k),(K,j_k)}
\end{pmatrix}.$$

where $\rho_{(k,j),(k^\star,j^\star)}$ equals one of the following: If $k = k^\star$ and $j = j^\star$ then $\rho_{(k,i),(k^\star,j^\star)} = 1$; If $k = k^\star$ and $j < j^\star$ then

$$\rho_{(k,j),(k^\star,j^\star)} = \frac{\sqrt{r_{k,j^\star}^{-1} + (r_{0,k,j^\star} - r(k))^{-1}}}{\sqrt{r_{k,j}^{-1} + (r_{0,k,j} - r(k))^{-1}}};$$

and if $k \neq k^\star$ where $r(k) < r(k^\star)$ then

$$\rho_{(k,j),(k^\star,j^\star)} = \max\left[0, \left(\sqrt{r_{k,j}^{-1} + (r_{0,k,j} - r(k))^{-1}}\sqrt{r_{k^\star,j^\star}^{-1} + (r_{0,k^\star,j^\star} - r(k^\star))^{-1}}\right)^{-1} \left(\frac{\min[r_{0,k,j} - r(k^\star), r_{0,k^\star,j^\star} - r(k^\star)]}{[r_{0,k,j} - r(k)][r_{0,k^\star,j^\star} - r(k^\star)]}\right)\right].$$

© 2010 WILEY-VCH Verlag GmbH & Co. KGaA, Weinheim

4 Disjunctive power

As discussed in Section 2.3 of the main paper the disjunctive power is the probability of taking at least one treatment forward. The disjunctive power can therefore be calculated in a very similar way to the FWER, as done in Section 2.2 and the Supporting Information Section 2, as here we want the probability of rejecting any null hypotheses $H_{01},\dots,H_{0K}$. Therefore if $\mu_k-\mu_0=\theta_k$ for $k=1,\dots,K$, the event that $H_{01},\dots,H_{0K}$ all fail to be rejected is equivalent to $\bar{R}_K(\Theta)$. The disjunctive power (P_d) for given $\Theta=(\theta_1,\dots,\theta_K)$ is:

$$P_d = 1-P(\bar{R}_K(\Theta)) = 1-\sum_{\substack{j_k=1\\ k=1,2,\dots,K}}^{J_k}\Phi(\mathbf{L_{j_k}}(\Theta),\mathbf{U_{j_k}}(\Theta),\Sigma_{\mathbf{j_k}}),$$

where $\mathbf{U_{j_k}}(\Theta)=(U_{1,j_1}(\theta_1),\dots,U_{K,j_K}(\theta_K))$ and $\mathbf{L_{j_k}}(\Theta)=(L_{1,j_1}(\theta_1),\dots,L_{K,j_K}(\theta_K))$ with $U_{k,j_k}(\theta_k)$ and $L_{k,j_k}(\theta_k)$ equalling

$$L_{k,j_k}(\theta_k) = (l_{k,1}-\frac{\theta_k}{\sqrt{I_{k,1}}},\dots,l_{k,j_k-1}-\frac{\theta_k}{\sqrt{I_{k,j_k-1}}},-\infty),$$
$$U_{k,j_k}(\theta_k) = (u_{k,1}-\frac{\theta_k}{\sqrt{I_{k,1}}},\dots,u_{k,j_k-1}-\frac{\theta_k}{\sqrt{I_{k,j_k-1}}},l_{k,j_k}-\frac{\theta_k}{\sqrt{I_{k,j_k}}}).$$

The correlation matrix $\Sigma_{\mathbf{j_k}}$ is the same as that given for FWER in Equation (3) of the main paper.

5 Proof of Theorem 2.1

Theorem 5.1 *For any* Θ, P(*reject all* H_{0k} *for which* $\theta_k\geq\theta'|\Theta)\geq P$(*reject all* H_{0k} *for which* $\theta_k\geq\theta'|\Theta')$.

Proof. For any $\epsilon_k<0$,

$$\bigcup_{j_k=1}^{J_k}\left[\left(\bigcap_{j=1}^{j_k-1}B_{k,j}(\theta_k+\epsilon_k)\right)\cap C_{k,j_k}(\theta_k+\epsilon_k)\right]\subseteq\bigcup_{j_k=1}^{J_k}\left[\left(\bigcap_{j=1}^{j_k-1}B_{k,j}(\theta_k)\right)\cap C_{k,j_k}(\theta_k)\right].$$

Take any

$$w=(Z_{k,1},\dots,Z_{k,J_k})\in\bigcup_{j_k=1}^{J_k}\left[\left(\bigcap_{j=1}^{j_k-1}B_{k,j}(\theta_k+\epsilon_k)\right)\cap C_{k,j_k}(\theta_k+\epsilon_k)\right].$$

For some $j_k\in\{1,\dots,J_k\}$, for which $Z_{k,j_k}\in C_{k,j_k}(\theta_k+\epsilon_k)$ and $Z_{k,j}\in B_{k,j}(\theta_k+\epsilon_k)$ for $j=1,\dots,j_k-1$. $Z_{k,j_k}\in C_{k,j_k}(\theta_k+\epsilon_k)$ implies that $Z_{k,j_k}\in C_{k,j_k}(\theta_k)$. Furthermore $Z_{k,j_k}\in B_{k,j_k}(\theta_k+\epsilon_k)$ implies that $Z_{k,j_k}\in B_{k,j_k}(\theta_k)\cup C_{k,j_k}(\theta_k)$ for some $j=1,\dots,j_k-1$. Therefore,

$$w\in\bigcup_{j_k=1}^{J_k}\left[\left(\bigcap_{j=1}^{j_k-1}B_{k,j}(\theta_k)\right)\cap C_{k,j_k}(\theta_k)\right].$$

Next suppose for any $m_1,\dots,m_K$ where $m_1\in\{1,\dots,K\}$ and $m_k\in\{1,\dots,K\}\backslash\{m_1,\dots,m_{k-1}\}$ with $\theta_{m_1},\dots,\theta_{m_l}\geq\theta'$ and $\theta_{m_{l+1}},\dots,\theta_{m_K}<\theta'$. Let $\Theta_l=(\theta_{m_1},\dots,\theta_{m_l})$. Then

$$\begin{aligned}P(\text{reject all } H_{0k} \text{ for which } \theta_k\geq\theta'|\Theta) &= P(\bar{W}_l(\Theta_l))\\ &\geq P(\bar{W}_l(\Theta'))\\ &\geq P(\bar{W}_k(\Theta'))\\ &= P(\text{reject all } H_{0k} \text{ for which } \theta_k\geq\theta'|\Theta').\end{aligned}$$

□

© 2010 WILEY-VCH Verlag GmbH & Co. KGaA, Weinheim

Table S5 Maximum sample size of the platform designs and the separate trials designs for different values of $n(2)$.

$n(2)$	$\max(N)$ for pairwise power design		
	Platform design	Separate trials with FWER control	Separate trials without FWER control
0	456	616	520
50	506	616	520
100	562	616	520
150	612	616	520
$n(2)$	$\max(N)$ for conjunctive power design		
	Platform design	Separate trials with FWER control	Separate trials without FWER control
0	558	784	680
50	620	784	680
100	676	784	680
150	732	784	680

6 Expected sample size when allowing pauses in recruitment

To calculate the expected sample size when allowing for pauses in recruitment for the control when there is no active treatments we build on Equation (9). The probability of each outcome remains the same ($Q_{\mathbf{j_k},\mathbf{q_k}}$). The total sample size associated with each $Q_{\mathbf{j_k},\mathbf{q_k}}$ changes. We define this as $N'_{\mathbf{j_k},\mathbf{q_k}}$. To calculate $N'_{\mathbf{j_k},\mathbf{q_k}}$ we set it so that the first active treatment begins the trial at the start (so $n(1) = 0$) and that each treatment is added in sequence, so treatment k is at the same time or after treatment $k-1$ (so $n(k-1) \leq n(k)$ for all $k = 2, \dots K$). To calculate $N'_{\mathbf{j_k},\mathbf{q_k}}$ we need to remove any period where just the control treatment is recruited, so

$$N'_{\mathbf{j_k},\mathbf{q_k}} = \left(\sum_{k=1}^{K} n_{k,j_k} \right) + \max_{k \in 1,\dots K} (n_{0,k,j_k}) - \sum_{k=2}^{K} \max[0, n(k) - \max_{i \in 1,\dots k} (n_{0,i,j_i})].$$

The expected sample size when allowing for pauses in recruitment when there are no active treatments is

$$E(N|\Theta) = \sum_{\substack{j_k=1 \\ k=1,2,\dots,K}}^{J_k} \sum_{\substack{q_k \in \{0,1\} \\ k=1,2,\dots,K}} Q_{\mathbf{j_k},\mathbf{q_k}} N'_{\mathbf{j_k},\mathbf{q_k}}.$$

7 Table of maximum sample sizes based on the results given in Section 3.3

Table S5 gives the maximum sample size for the pairwise power designs and conjunctive power designs using the same results as given in Figure 1 and Figure 2 of the main manuscript.

8 O'Brien and Fleming boundaries and the Pocock boundaries

Using the trial setting introduced in Section 3 the O'Brien and Fleming boundaries (O'Brien and Fleming, 1979) with the futility boundaries equal to zero for $j < J_K$, to remove the symmetric boundaries, which may be too stringent (Magirr et al., 2012) give the stopping boundaries

$$\begin{pmatrix} U_1 \\ U_2 \end{pmatrix} = \begin{pmatrix} 3.166 & 2.239 \\ 3.166 & 2.239 \end{pmatrix}, \quad \begin{pmatrix} L_1 \\ L_2 \end{pmatrix} = \begin{pmatrix} 0 & 2.239 \\ 0 & 2.239 \end{pmatrix}.$$

© 2010 WILEY-VCH Verlag GmbH & Co. KGaA, Weinheim

Table S6 Operating characteristics of the proposed designs under different values of θ_1 and θ_2, for both control of pairwise power and of conjunctive power, when the proposed designs use O'Brien and Fleming boundaries (O'Brien and Fleming, 1979) with futility boundaries equal to zero.

θ_1	θ_2	$P_{PW,1}$	$P_{PW,2}$	P_C	P_D	$\max(N)$	$E(N\|\theta_1,\theta_2)$
**Design for pairwise power**							
θ'	θ'	0.806	0.806	0.671	0.941	490	452.3
θ'	0	0.806	0.013	0.806	0.807	490	407.3
θ'	$-\infty$	0.806	0	0.806	0.806	490	337.4
0	θ'	0.013	0.806	0.806	0.807	490	429.7
0	0	0.013	0.013	1	0.025	490	384.8
$-\infty$	θ'	0	0.806	0.806	0.806	490	394.8
**Design for conjunctive power**							
θ_1	θ_2	$P_{PW,1}$	$P_{PW,2}$	P_C	P_D	$\max(N)$	$E(N\|\theta_1,\theta_2)$
θ'	θ'	0.889	0.889	0.801	0.977	609	545.5
θ'	0	0.889	0.013	0.889	0.889	609	500.7
θ'	$-\infty$	0.889	0	0.889	0.889	609	413.8
0	θ'	0.013	0.889	0.889	0.889	609	523.1
0	0	0.013	0.013	1	0.025	609	478.3
$-\infty$	θ'	0	0.889	0.889	0.889	609	479.6

When the focus is on ensuring that the pairwise power is greater than 80% the sample sizes are

$$\begin{pmatrix} n_{1,1} & n_{1,2} \\ n_{2,1} & n_{2,2} \end{pmatrix} = \begin{pmatrix} 70 & 140 \\ 70 & 140 \end{pmatrix}, \quad \begin{pmatrix} n_{0,1,1} & n_{0,1,2} \\ n_{0,2,1} & n_{0,2,2} \end{pmatrix} = \begin{pmatrix} 70 & 140 \\ 140 & 210 \end{pmatrix}. \quad \begin{pmatrix} n(1) \\ n(2) \end{pmatrix} = \begin{pmatrix} 0 \\ 70 \end{pmatrix}.$$

When ensuring that the conjunctive power is greater than 80% the sample sizes are

$$\begin{pmatrix} n_{1,1} & n_{1,2} \\ n_{2,1} & n_{2,2} \end{pmatrix} = \begin{pmatrix} 87 & 174 \\ 87 & 174 \end{pmatrix}, \quad \begin{pmatrix} n_{0,1,1} & n_{0,1,2} \\ n_{0,2,1} & n_{0,2,2} \end{pmatrix} = \begin{pmatrix} 87 & 174 \\ 174 & 261 \end{pmatrix}. \quad \begin{pmatrix} n(1) \\ n(2) \end{pmatrix} = \begin{pmatrix} 0 \\ 87 \end{pmatrix}.$$

Table S6 shows the results for different values of θ_1 and θ_2 when the conjunctive power is greater than 80% and when the pairwise power is greater than 80%.

The Pocock boundaries (Pocock, 1977) with the futility boundaries equal to zero for $j < J_K$, to remove the symmetric boundaries, which may be too stringent (Magirr et al., 2012) give the stopping boundaries

$$\begin{pmatrix} U_1 \\ U_2 \end{pmatrix} = \begin{pmatrix} 2.440 & 2.440 \\ 2.440 & 2.440 \end{pmatrix}, \quad \begin{pmatrix} L_1 \\ L_2 \end{pmatrix} = \begin{pmatrix} 0 & 2.440 \\ 0 & 2.440 \end{pmatrix}.$$

When the focus is on ensuring that the pairwise power is greater than 80% the sample sizes are:

$$\begin{pmatrix} n_{1,1} & n_{1,2} \\ n_{2,1} & n_{2,2} \end{pmatrix} = \begin{pmatrix} 76 & 152 \\ 76 & 152 \end{pmatrix}, \quad \begin{pmatrix} n_{0,1,1} & n_{0,1,2} \\ n_{0,2,1} & n_{0,2,2} \end{pmatrix} = \begin{pmatrix} 76 & 152 \\ 152 & 228 \end{pmatrix}. \quad \begin{pmatrix} n(1) \\ n(2) \end{pmatrix} = \begin{pmatrix} 76 \\ 152 \end{pmatrix}.$$

When ensuring that the conjunctive power is greater than 80% the sample sizes are:

$$\begin{pmatrix} n_{1,1} & n_{1,2} \\ n_{2,1} & n_{2,2} \end{pmatrix} = \begin{pmatrix} 95 & 190 \\ 95 & 190 \end{pmatrix}, \quad \begin{pmatrix} n_{0,1,1} & n_{0,1,2} \\ n_{0,2,1} & n_{0,2,2} \end{pmatrix} = \begin{pmatrix} 95 & 190 \\ 190 & 285 \end{pmatrix}. \quad \begin{pmatrix} n(1) \\ n(2) \end{pmatrix} = \begin{pmatrix} 0 \\ 95 \end{pmatrix}.$$

Table S7 shows the results for different values of θ_1 and θ_2 when the conjunctive power is greater than 80% and when the pairwise power is greater than 80%.

© 2010 WILEY-VCH Verlag GmbH & Co. KGaA, Weinheim

Table S7 Operating characteristics of the proposed designs under different values of θ_1 and θ_2, for both control of pairwise power and of conjunctive power, when the proposed designs use Pocock boundaries (Pocock, 1977) with futility boundaries equal to zero.

Design for pairwise power

θ_1	θ_2	$P_{PW,1}$	$P_{PW,2}$	P_C	P_D	$\max(N)$	$E(N\|\theta_1,\theta_2)$
θ'	θ'	0.802	0.802	0.662	0.941	532	429.3
θ'	0	0.802	0.013	0.802	0.802	532	420.6
θ'	$-\infty$	0.802	0	0.802	0.802	532	345.7
0	θ'	0.013	0.802	0.802	0.803	532	424.9
0	0	0.013	0.013	1	0.025	532	416.3
$-\infty$	θ'	0	0.802	0.802	0.802	532	387.5
**Design for conjunctive power**							
θ_1	θ_2	$P_{PW,1}$	$P_{PW,2}$	P_C	P_D	$\max(N)$	$E(N\|\theta_1,\theta_2)$
θ'	θ'	0.889	0.889	0.801	0.978	665	507.6
θ'	0	0.889	0.013	0.889	0.890	665	516.1
θ'	$-\infty$	0.889	0	0.889	0.889	665	422.5
0	θ'	0.013	0.889	0.889	0.890	665	511.9
0	0	0.013	0.013	1	0.025	665	520.4
$-\infty$	θ'	0	0.889	0.889	0.889	665	465.1

9 Non-binding stopping boundaries

Using the trial setting introduced in Section 3 the triangular boundaries (Whitehead, 1997) with non-binding futility boundaries for the type I error, are

$$\begin{pmatrix} U_1 \\ U_2 \end{pmatrix} = \begin{pmatrix} 2.517 & 2.373 \\ 2.517 & 2.373 \end{pmatrix}, \quad \begin{pmatrix} L_1 \\ L_2 \end{pmatrix} = \begin{pmatrix} 0.839 & 2.373 \\ 0.839 & 2.373 \end{pmatrix}.$$

When the focus is on ensuring that the pairwise power is greater than 80% the sample sizes are:

$$\begin{pmatrix} n_{1,1} & n_{1,2} \\ n_{2,1} & n_{2,2} \end{pmatrix} = \begin{pmatrix} 77 & 154 \\ 77 & 154 \end{pmatrix}, \quad \begin{pmatrix} n_{0,1,1} & n_{0,1,2} \\ n_{0,2,1} & n_{0,2,2} \end{pmatrix} = \begin{pmatrix} 77 & 154 \\ 154 & 231 \end{pmatrix}. \quad \begin{pmatrix} n(1) \\ n(2) \end{pmatrix} = \begin{pmatrix} 0 \\ 77 \end{pmatrix}.$$

When ensuring that the conjunctive power is greater than 80% the sample sizes are:

$$\begin{pmatrix} n_{1,1} & n_{1,2} \\ n_{2,1} & n_{2,2} \end{pmatrix} = \begin{pmatrix} 97 & 194 \\ 97 & 194 \end{pmatrix}, \quad \begin{pmatrix} n_{0,1,1} & n_{0,1,2} \\ n_{0,2,1} & n_{0,2,2} \end{pmatrix} = \begin{pmatrix} 97 & 194 \\ 194 & 291 \end{pmatrix}. \quad \begin{pmatrix} n(1) \\ n(2) \end{pmatrix} = \begin{pmatrix} 0 \\ 97 \end{pmatrix}.$$

Table S8 shows the results for different values of θ_1 and θ_2 when the conjunctive power is greater than 80% and when the pairwise power is greater than 80%. As can be seen in these results, unlike in Table 2, the disjunctive power no longer equals the target of 2.5% when $\theta_1, \theta_2 = 0$. This is because this is the FWER if one did use the lower boundaries for futility. Without these lower bounds the FWER is 2.5%. This is the same for the PWER when looking at the pairwise power when θ_1 or θ_2 equals 0.

The results when using non-binding O'Brien and Fleming boundaries (O'Brien and Fleming, 1979) with the futility boundaries equal to zero for $j < J_K$, to remove the symmetric boundaries, which may be too stringent (Magirr et al., 2012) give the stopping boundaries

$$\begin{pmatrix} U_1 \\ U_2 \end{pmatrix} = \begin{pmatrix} 3.168 & 2.400 \\ 3.168 & 2.400 \end{pmatrix}, \quad \begin{pmatrix} L_1 \\ L_2 \end{pmatrix} = \begin{pmatrix} 0 & 2.400 \\ 0 & 2.400 \end{pmatrix}.$$

When the focus is on ensuring that the pairwise power is greater than 80% the sample sizes are:

$$\begin{pmatrix} n_{1,1} & n_{1,2} \\ n_{2,1} & n_{2,2} \end{pmatrix} = \begin{pmatrix} 70 & 140 \\ 70 & 140 \end{pmatrix}, \quad \begin{pmatrix} n_{0,1,1} & n_{0,1,2} \\ n_{0,2,1} & n_{0,2,2} \end{pmatrix} = \begin{pmatrix} 70 & 140 \\ 140 & 210 \end{pmatrix}. \quad \begin{pmatrix} n(1) \\ n(2) \end{pmatrix} = \begin{pmatrix} 70 \\ 140 \end{pmatrix}.$$

© 2010 WILEY-VCH Verlag GmbH & Co. KGaA, Weinheim

Table S8 Operating characteristics of the proposed designs under different values of θ_1 and θ_2, for both control of pairwise power and of conjunctive power, when the proposed designs use triangular boundaries (Whitehead, 1997) with non-binding futility boundaries for the type I error.

Design for pairwise power

θ_1	θ_2	$P_{PW,1}$	$P_{PW,2}$	P_C	P_D	$\max(N)$	$E(N\|\theta_1,\theta_2)$
θ'	θ'	0.802	0.802	0.663	0.942	539	426.6
θ'	0	0.802	0.012	0.802	0.803	539	377.5
θ'	$-\infty$	0.802	0	0.802	0.802	539	347.5
0	θ'	0.012	0.802	0.802	0.804	539	402.0
0	0	0.012	0.012	1	0.024	539	353.0
$-\infty$	θ'	0	0.802	0.802	0.802	539	387.0

Design for conjunctive power

θ_1	θ_2	$P_{PW,1}$	$P_{PW,2}$	P_C	P_D	$\max(N)$	$E(N\|\theta_1,\theta_2)$
θ'	θ'	0.891	0.891	0.803	0.979	679	513.9
θ'	0	0.891	0.012	0.891	0.891	679	467.7
θ'	$-\infty$	0.891	0	0.891	0.891	679	430.0
0	θ'	0.012	0.891	0.891	0.891	679	490.8
0	0	0.012	0.012	1	0.024	679	444.7
$-\infty$	θ'	0	0.891	0.891	0.891	679	471.9

Table S9 Operating characteristics of the proposed designs under different values of θ_1 and θ_2, for both control of pairwise power and of conjunctive power, when the proposed designs use non-binding O'Brien and Fleming boundaries (O'Brien and Fleming, 1979) with futility boundaries equal to zero.

Design for pairwise power

θ_1	θ_2	$P_{PW,1}$	$P_{PW,2}$	P_C	P_D	$\max(N)$	$E(N\|\theta_1,\theta_2)$
θ'	θ'	0.806	0.806	0.670	0.942	490	452.4
θ'	0	0.806	0.013	0.806	0.807	490	407.3
θ'	$-\infty$	0.806	0	0.806	0.806	490	337.4
0	θ'	0.013	0.806	0.806	0.807	490	429.7
0	0	0.013	0.013	1	0.025	490	384.8
$-\infty$	θ'	0	0.806	0.806	0.806	490	394.8

Design for conjunctive power

θ_1	θ_2	$P_{PW,1}$	$P_{PW,2}$	P_C	P_D	$\max(N)$	$E(N\|\theta_1,\theta_2)$
θ'	θ'	0.892	0.892	0.806	0.978	616	550.8
θ'	0	0.892	0.013	0.892	0.892	616	506.1
θ'	$-\infty$	0.892	0	0.892	0.892	616	418.3
0	θ'	0.013	0.892	0.892	0.892	616	528.5
0	0	0.013	0.013	1	0.025	616	483.77
$-\infty$	θ'	0	0.892	0.892	0.892	616	484.5

When ensuring that the conjunctive power is greater than 80% the sample sizes are:

$$\begin{pmatrix} n_{1,1} & n_{1,2} \\ n_{2,1} & n_{2,2} \end{pmatrix} = \begin{pmatrix} 88 & 176 \\ 88 & 176 \end{pmatrix}, \quad \begin{pmatrix} n_{0,1,1} & n_{0,1,2} \\ n_{0,2,1} & n_{0,2,2} \end{pmatrix} = \begin{pmatrix} 88 & 176 \\ 176 & 264 \end{pmatrix}. \quad \begin{pmatrix} n(1) \\ n(2) \end{pmatrix} = \begin{pmatrix} 0 \\ 88 \end{pmatrix}.$$

Table S9 shows the results for different values of θ_1 and θ_2 when the conjunctive power is greater than 80% and when the pairwise power is greater than 80%.

The results when using non-binding Pocock boundaries (Pocock, 1977) with the futility boundaries equal to zero for $j < J_K$, to remove the symmetric boundaries, which may be too stringent (Magirr et al.,

© 2010 WILEY-VCH Verlag GmbH & Co. KGaA, Weinheim

Table S10 Operating characteristics of the proposed designs under different values of θ_1 and θ_2, for both control of pairwise power and of conjunctive power, when the proposed designs use non-binding Pocock boundaries (Pocock, 1977) with futility boundaries equal to zero.

**Design for pairwise power**							
θ_1	θ_2	$P_{PW,1}$	$P_{PW,2}$	P_C	P_D	$\max(N)$	$E(N\|\theta_1,\theta_2)$
θ'	θ'	0.801	0.801	0.661	0.940	532	429.6
θ'	0	0.801	0.013	0.801	0.801	532	420.7
θ'	$-\infty$	0.801	0	0.801	0.801	532	345.9
0	θ'	0.013	0.801	0.801	0.802	532	425.2
0	0	0.013	0.013	1	0.025	532	416.3
$-\infty$	θ'	0	0.801	0.801	0.801	532	387.7
**Design for conjunctive power**							
θ_1	θ_2	$P_{PW,1}$	$P_{PW,2}$	P_C	P_D	$\max(N)$	$E(N\|\theta_1,\theta_2)$
θ'	θ'	0.892	0.892	0.805	0.979	672	511.9
θ'	0	0.892	0.013	0.892	0.893	672	521.2
θ'	$-\infty$	0.892	0	0.892	0.892	672	426.6
0	θ'	0.013	0.892	0.892	0.893	672	516.6
0	0	0.013	0.013	1	0.025	672	525.9
$-\infty$	θ'	0	0.892	0.892	0.892	672	469.3

2012) give the stopping boundaries

$$\begin{pmatrix} U_1 \\ U_2 \end{pmatrix} = \begin{pmatrix} 2.444 & 2.444 \\ 2.444 & 2.444 \end{pmatrix}, \quad \begin{pmatrix} L_1 \\ L_2 \end{pmatrix} = \begin{pmatrix} 0 & 2.444 \\ 0 & 2.444 \end{pmatrix}.$$

When the focus is on ensuring that the pairwise power is greater than 80% the sample sizes are:

$$\begin{pmatrix} n_{1,1} & n_{1,2} \\ n_{2,1} & n_{2,2} \end{pmatrix} = \begin{pmatrix} 76 & 152 \\ 76 & 152 \end{pmatrix}, \quad \begin{pmatrix} n_{0,1,1} & n_{0,1,2} \\ n_{0,2,1} & n_{0,2,2} \end{pmatrix} = \begin{pmatrix} 76 & 152 \\ 152 & 228 \end{pmatrix}. \quad \begin{pmatrix} n(1) \\ n(2) \end{pmatrix} = \begin{pmatrix} 76 \\ 152 \end{pmatrix}.$$

When ensuring that the conjunctive power is greater than 80% the sample sizes are:

$$\begin{pmatrix} n_{1,1} & n_{1,2} \\ n_{2,1} & n_{2,2} \end{pmatrix} = \begin{pmatrix} 96 & 192 \\ 96 & 192 \end{pmatrix}, \quad \begin{pmatrix} n_{0,1,1} & n_{0,1,2} \\ n_{0,2,1} & n_{0,2,2} \end{pmatrix} = \begin{pmatrix} 96 & 192 \\ 192 & 288 \end{pmatrix}. \quad \begin{pmatrix} n(1) \\ n(2) \end{pmatrix} = \begin{pmatrix} 0 \\ 96 \end{pmatrix}.$$

Table S10 shows the results for different values of θ_1 and θ_2 when the conjunctive power is greater than 80% and when the pairwise power is greater than 80%.

10 Plots based on the results from Section 3.5 for the two and three stage designs

The plots for the 2 stage and 3 stage example trials as given in Table 3 are shown in Figure S1 and Figure S2. These plots are similar to the once seen in Figure 1 and 2 of the main paper. The y-axis gives the sample size for the trial. The x-axis gives the amount of control patients recruited between each active treatment being added ($n(k) - n(k-1)$). Plotted on the graph is the maximum sample size and the expected sample size under the different configurations considered in Table 3. Figure S1 gives the plots when the pairwise power is controlled at 80% and Figure S2 gives the plots when the conjunctive power is controlled at 80%. As can be seen in Figure S2 there are times where some of the lines are at the same point. This is caused when separate trials become better than running the proposed platform trial being at the same point for multiple different Θ, as seen in Table 3, therefore the lines overlap.

© 2010 WILEY-VCH Verlag GmbH & Co. KGaA, Weinheim

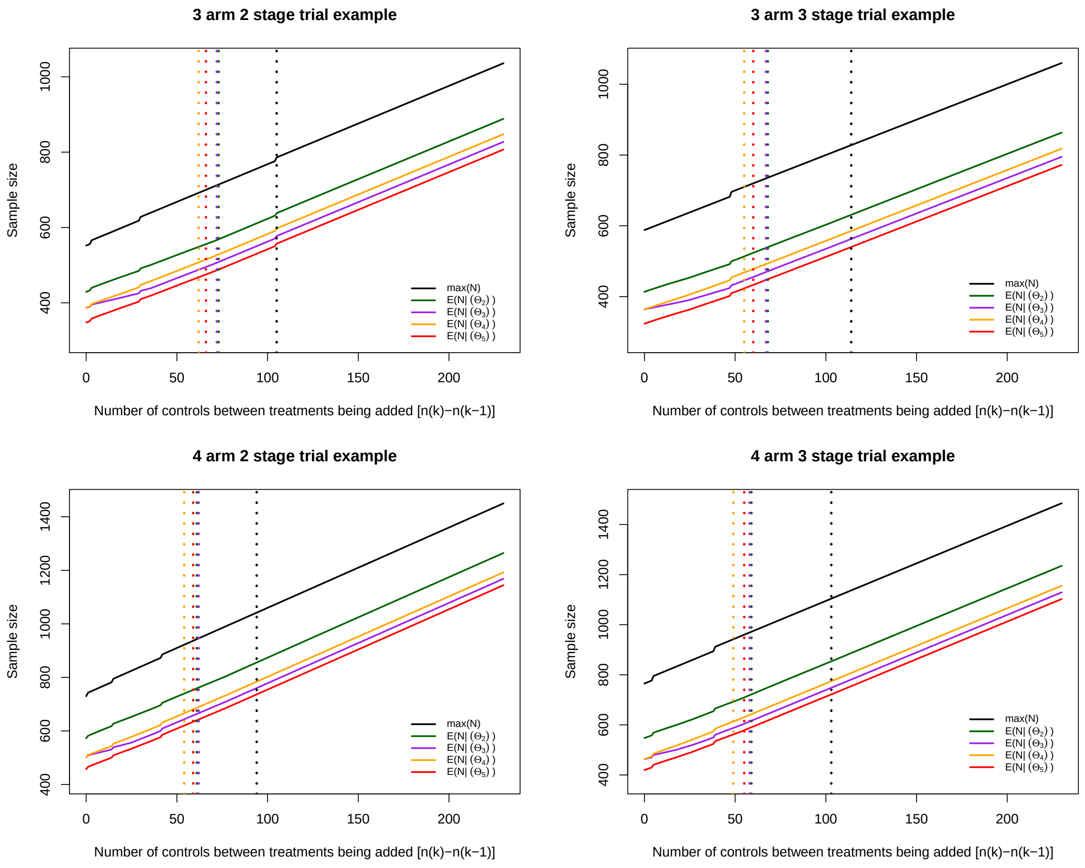

Figure S1 The maximum sample size and the expected sample size under different Θ depending on the value $n(k) - n(k-1)$, for the pairwise power control of 80% and FWER of 5% one-sided. The dash vertical lines correspond to the points where the maximum or expected sample size of the trial is now greater than running separate trials which each have type I error control of 2.5% one-sided.

Acknowledgements This report is independent research supported by the National Institute for Health Research (NIHR300576). The views expressed in this publication are those of the authors and not necessarily those of the NHS, the National Institute for Health Research or the Department of Health and Social Care (DHSC). TJ and PM also received funding from UK Medical Research Council (MC_UU_00040/03, MC_UU_00002/14 and MC_UU_00002/19). This paper is based on work completed while PG was part of the EPSRC funded STOR-i centre for doctoral training (EP/S022252/1). For the purpose of open access, the author has applied a Creative Commons Attribution (CC BY) licence to any Author Accepted Manuscript version arising. We would like to thank the two reviewers, the Special Issue Editor and the Editor for their useful comments and suggestions.

Conflict of Interest

The authors declare no potential conflict of interests. Alun Bedding is a shareholder of Roche Products Ltd.

© 2010 WILEY-VCH Verlag GmbH & Co. KGaA, Weinheim

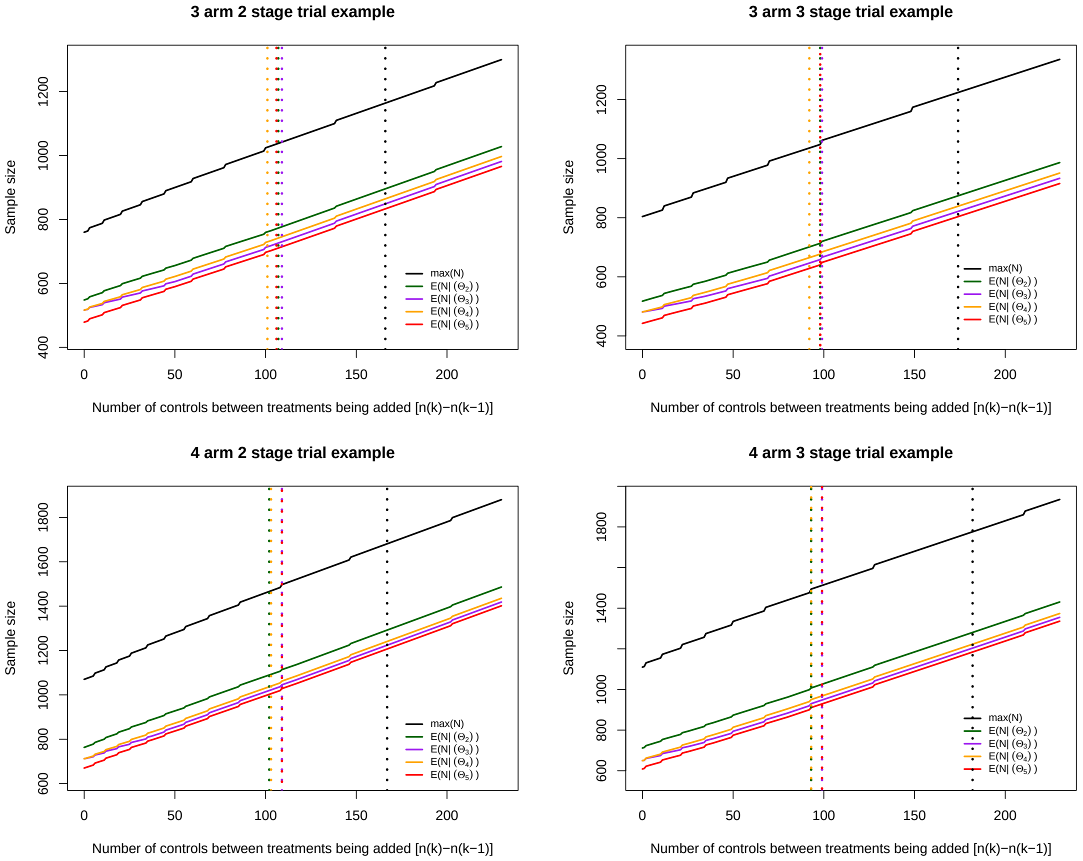

Figure S2 The maximum sample size and the expected sample size under different Θ depending on the value $n(k) - n(k-1)$, for the conjunctive power control of 80% and FWER of 5% one-sided. The dash vertical lines correspond to the points where the maximum or expected sample size of the trial is now greater than running separate trials which each have type I error control of 2.5% one-sided.

References

Greenstreet, P., Jaki, T., Bedding, A., Harbron, C., and Mozgunov, P. (2024). A multi-arm multi-stage platform design that allows preplanned addition of arms while still controlling the family-wise error. *Statistics in Medicine*.

Magirr, D., Jaki, T., and Whitehead, J. (2012). A generalized Dunnett test for multi-arm multi-stage clinical studies with treatment selection. *Biometrika*, 99(2):494–501.

O'Brien, P. C. and Fleming, T. R. (1979). A Multiple Testing Procedure for Clinical Trials. *Biometrics*, 35(3):549–556.

Pocock, S. J. (1977). Group Sequential Methods in the Design and Analysis of Clinical Trials. *Biometrika*, 64(2):191–199.

Whitehead, J. (1997). The Design and Analysis of Sequential Clinical Trials. *Biometrics*, 53(4):1564.

© 2010 WILEY-VCH Verlag GmbH & Co. KGaA, Weinheim
